# *In toto* light sheet fluorescence microscopy live imaging datasets of *Ceratitis capitata* embryonic development

**DOI:** 10.1038/s41597-022-01443-x

**Published:** 2022-06-15

**Authors:** Frederic Strobl, Marc F. Schetelig, Ernst H. K. Stelzer

**Affiliations:** 1 Physical Biology/Physikalische Biologie (IZN, FB 15). Buchmann Institute for Molecular Life Sciences (BMLS). Cluster of Excellence Frankfurt – Macromolecular Complexes (CEF – MC), Goethe-Universität – Frankfurt am Main (Campus Riedberg), Max-von-Laue-Straße 15, D-60438 Frankfurt am Main, Germany; 2 Justus-Liebig-Universität Gießen. Department of Insect Biotechnology in Plant Protection, Winchesterstraße 2, D-35394 Gießen, Germany

**Keywords:** Morphogenesis, Fluorescence imaging, Embryogenesis, Light-sheet microscopy

## Abstract

The Mediterranean fruit fly (medfly), *Ceratitis capitata*, is an important model organism in biology and agricultural research with high economic relevance. However, information about its embryonic development is still sparse. We share nine long-term live imaging datasets acquired with light sheet fluorescence microscopy (484.5 h total recording time, 373 995 images, 256 Gb) with the scientific community. Six datasets show the embryonic development *in toto* for about 60 hours at 30 minutes intervals along four directions in three spatial dimensions, covering approximately 97% of the entire embryonic development period. Three datasets focus on germ cell formation and head involution. All imaged embryos hatched morphologically intact. Based on these data, we suggest a two-level staging system that functions as a morphogenetic framework for upcoming studies on medfly. Our data supports research on wild-type or aberrant morphogenesis, quantitative analyses, comparative approaches to insect development as well as studies related to pest control. Further, they can be used to test advanced image processing approaches or to train machine learning algorithms and/or neuronal networks.

## Abstract (summary)

**Table:** 

Design Type(s)	non-invasive long-term fluorescence live imaging
Measurement Type(s)	three-dimensional fluorophore distribution over time in a developing organism
Technology Type(s)	digital scanned laser light sheet fluorescence microscopy (DSLM)
Factor Type(s)	
Sample Characteristics	*Ceratitis capitata*

**Table:** 

Measurement(s)	three-dimensional fluorophore distribution as a function of time
Technology Type(s)	light sheet fluorescence microscopy
Sample Characteristic - Organism	Ceratitis capitata

## Background & Summary

In insect developmental biology, comparative approaches shed light on the broad variety of developmental strategies and contribute to our understanding of the evolution of development^1^. To study embryonic morphogenesis on the cellular and subcellular level, light sheet fluorescence microscopy (LSFM) became the method of choice. It allows non-invasive live imaging of millimeter-sized specimens for time periods up to several days^2–8^ and has already been successfully applied to characterize the embryonic morphogenesis of several insect species such as the fruit fly *Drosophila melanogaster*^9–15^, the scuttle fly *Megaselia abdita*^16^ and the red flour beetle *Tribolium castaneum*^17–20^. Due to the intrinsic properties of LSFM^21^, *e.g*., a high signal-to-noise ratio and good depth penetration in conjunction with nearly no photobleaching and phototoxicity, the acquired datasets typically provide a profound collection of high-quality images with excellent temporal and good three-dimensional spatial resolutions. The quality and quantity of the acquired data usually exceeds the requirements for the respective study, and in many cases, only a fraction of the data is analyzed. Thus, it is convenient to share these data as an open access resource with the scientific community, since carefully staged morphogenetic information support research on wild-type or aberrant development, enable quantitative analyses and foster comparative approaches. Further, systematically acquired image data can be used to train machine learning algorithms and/or neuronal networks and are thus a major step towards high-volume AI-based research in developmental biology.

During the past decades, the number of insect model organisms increased considerably^22,23^. The Mediterranean fruit fly (medfly), *Ceratitis capitata* (Wiedemann), which belongs to the Diptera order, is a highly invasive agricultural pest with high economic relevance and became an important model organism for basic as well as pest management-associated research^24^. Standard and advanced techniques, such as germline transformation^25,26^, site-specific recombination^27^, targeted gene editing^28,29^, and cryopreservation^30^, are established. Further, the medfly genome sequence, available since 2016^31^, was recently improved^32^. *D. melanogaster* and *C. capitata* are both members of the Schizophora section but belong to different families. Phylogenetic analyses have shown that they diverged approximately 80–100 million years ago^33–35^. Regarding their embryonic development, both genera share apomorphic characteristics such as reduced extra-embryonic membranes, *i.e*., they form and degrade only one dorsally located membrane, the amnioserosa^36^. The closest comprehensively examined model organism, *M. abdita*^37^, which is a member of the Aschiza section that diverged from *D. melanogaster* and *C. capitata* approximately 150 million years ago^38^, develops two extra-embryonic membranes^39–41^. Thus, *C. capitata* bridges the phylogenetic gap between *D. melanogaster* and *M. abdita* and complements the existing pool of Dipteran model organisms for evolutionary developmental biology research. Studies on various biological questions have already been published, such as spatiotemporal gene expression patterns^42,43^, transcriptomics^44–46^, oogenesis^47^, larval morphology^48^ and antennal lobe structure^49^ but no comprehensive morphogenetic data and staging system for embryonic development has been available.

Using the cobweb holder approach^21,50^, an easy-to-use mounting method for insect embryos in LSFM, we recorded nine live imaging datasets of nine individual medfly embryos (484.5 h total recording time, 373 995 images, 256 Gb). Six datasets show the embryonic development *in toto* at 30 minutes intervals along four directions in three spatial dimensions, covering approximately 97% of the entire embryonic development period. Since the embryos were recorded at room temperature (23 ± 1 °C), development lasted for about 60 h^51^. The remaining three datasets focus on specific processes, such as germ cell formation and head involution. All imaged specimens hatched morphologically intact, and all but one developed into a healthy adult. Based on the acquired datasets, we established a morphogenesis-based two-level staging system that serves as a framework for future developmental studies in the medfly. We comprehensively quantify the temporal course of embryogenesis in both absolute terms as well as relative to total embryonic development and calculate the respective standard deviations for all time points. Taken together, our study provides the first long-term live imaging data of medfly embryonic development and thus contributes considerably to insect development biology, the comparative approach and pest management-associated research.

## Methods

### Transgenic medfly line and culture

This study used the *TREhs43-hid*^*Ala5*^_F1m2 transgenic medfly line, which expresses nuclear-localized EGFP under control of the *D. melanogaster* polyubiquitin promoter^52^. Medfly cultures, homozygous for the transgene, were kept at 25 °C, 50% relative humidity in a 12-h bright/12-h dark cycle (DR-36VL, Percival Scientific, Perry, IA, United States) in transparent acrylic boxes (approximately 15 × 15 × 20 cm) in groups of around 80 individuals. The plastic boxes had sideward openings that were covered with fine-meshed gaze to allow embryo deposition. Medflies were reared on a 2:1 mixture of refined sugar (524973, REWE Markt GmbH, Köln, Germany) to inactive dry yeast (62–106, Flystuff, San Diego, CA, USA) that was moistened with autoclaved tap water. Additionally, autoclaved tap water was provided on a wet tissue.

### Light sheet fluorescence microscopy

**Fig. 1:**
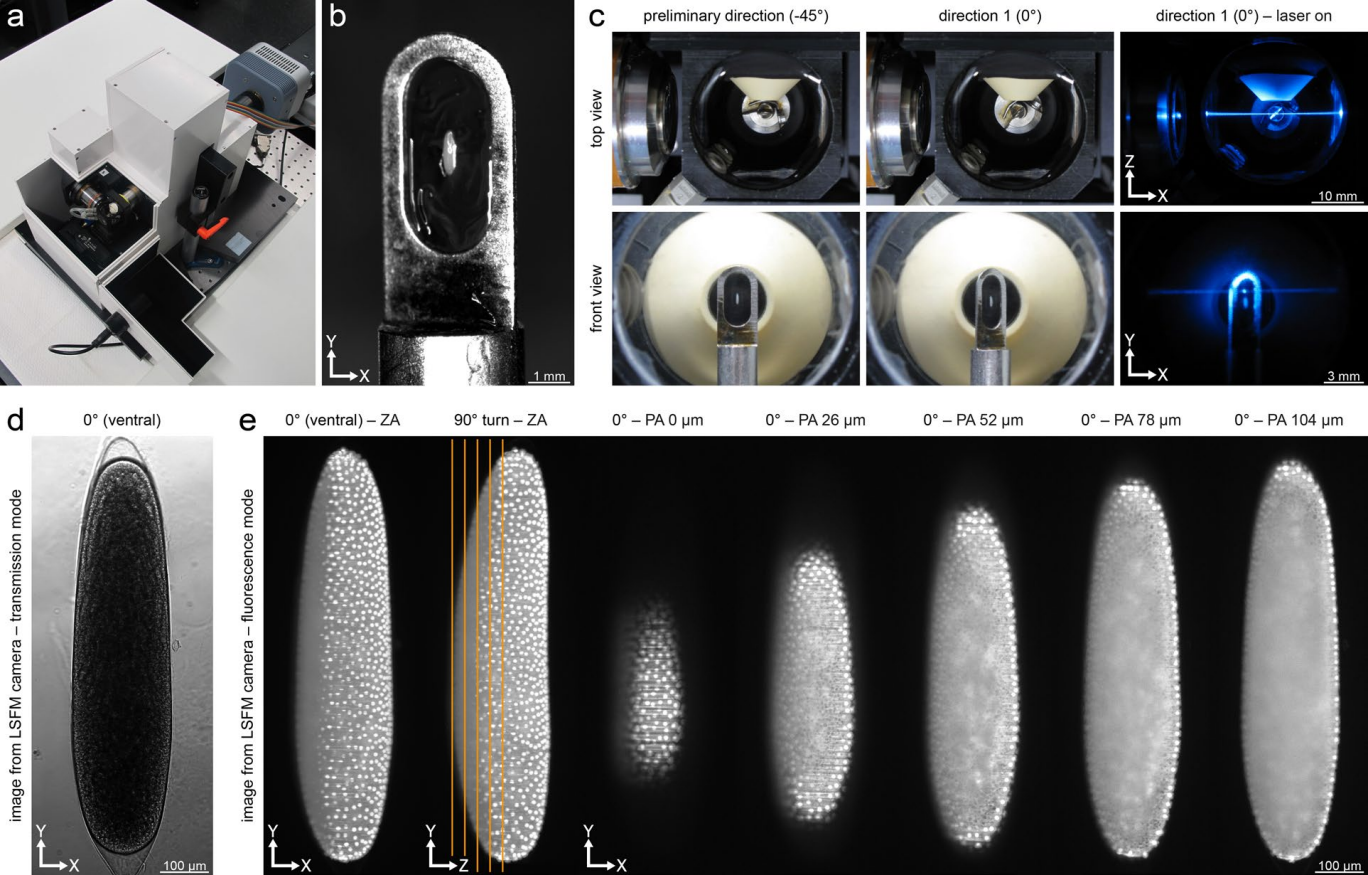
LSFM implementation and cobweb holder mounting method for medfly embryos. (**a**) Diagonal view of the sample chamber-based DSLM. (**b**) Cobweb holder with a medfly embryo embedded into an agarose film. (**c**) Top view (upper row) and front view (lower row) of the sample chamber. First the embryo is located and centered along the *x* and *y* axes in front of the detection objective (first column), defined as the preliminary direction (−45°). Subsequently, the embryo is rotated by 45° (second column), defined as direction 1 (0°). This setup allows the illumination of the embryo with a laser beam and the simultaneous acquisition of images through the detection objective (third column). (**d**) Transmission light image of a medfly embryo in the DSLM. (**e**) Representative fluorescence image data. Shown are *z* maximum projections along two orthogonal orientations (0° and 90°) and five planes at increasing depths (0 µm to 104 µm, *z* spacing 26 µm). In the 90° *z* maximum projections, the locations of the planes are indicated by orange lines. ZA, *z* maximum projection with image adjustment; PA, plane with image adjustment.

LSFM was implemented with a sample chamber-based digital scanned laser light sheet fluorescence microscope (DSLM, Fig. 1a)^21^, which generates a dynamic light sheet by rapidly scanning a Gaussian laser beam with a two-axes piezo-driven scanning mirror (M-116.DG, Physik Instrumente GmbH & Co KG, Karlsruhe, Germany). As the illumination light source, a 488 nm/60 mW diode laser (PhoxX 488-20, Omicron Laserprodukte GmbH, Rodgau-Dudenhofen, Germany) with a 488 nm cleanup filter (xX.F488, Omicron Laserprodukte GmbH, Rodgau-Dudenhofen, Germany) was used. Illumination was performed through a 2.5× NA 0.06 EC Epiplan-Neofluar objective (422320-9900-000, Carl Zeiss AG, Göttingen, Germany) and signal was collected either through a 10× NA 0.3 W N-Achroplan objective (420947-9900-000, Carl Zeiss AG, Göttingen, Germany) or through a 20× NA 0.5 W N-Achroplan objective (420957-9900-000, Carl Zeiss AG, Göttingen, Germany). In both setups, a 525/50 single-band bandpass filter (FF03-525/50-25, Semrock/AHF Analysentechnik AG, Tübingen, Germany) and a high-resolution CCD camera (Clara, Andor, Belfast, United Kingdom) were used for detection. Conventionally^21^, the illumination axis is defined as *x*, the rotation axis was defined as *y*, and the detection axis is defined as *z*. For convenience, *y* is parallel to the Earth’s gravitational axis. Axes are mentioned in the manuscript or indicated on figures whenever appropriate. The DSLM was further equipped with a 760-nm diode to acquire transmission light images. Three micro-translation stages (M-111.2DG, Physik Instrumente GmbH & Co KG, Karlsruhe, Germany) and a precision rotation stage (M-116.DG, Physik Instrumente GmbH & Co KG, Karlsruhe, Germany) were used for sample translation along *x*, *y* and *z* and rotation around *y*, respectively.

### Embryo collection and preparation

For embryo collection, medfly cultures were removed from the incubator. Old embryos were removed from the fine-meshed gaze and discarded. The cultures were given 10 min for embryo deposition at room temperature (23 ± 1 °C). All embryos (typically 5 to 20 per culture) laid in this time window were transferred to a 100 µm cell strainer (#352360, BD Biosciences, Heidelberg, Germany) with a paint brush. The embryos were moistened in PBS pH 7.4 (10010-023, Gibco Life Technologies GmbH, Darmstadt, Germany), dechorionated for 90 seconds in a 1:9 mixture of ~10% (vol/vol) sodium hypochlorite solution (425044-250 ML, Sigma Aldrich, Taufkirchen, Germany) and PBS and then washed twice in PBS for 60 seconds.

### Mounting using the cobweb holder

To keep the medfly embryos mechanically stable end ensure precise movement within the sample chamber during repeated movement and recording sequences (translation along *z* while recording, rotation around *y* followed by translation along *x*, *y* and *z* to reposition the embryo before acquisition of the next *z* stack) we used the cobweb holder mounting method^21,50^. The cobweb holder used in this study consists of a stainless-steel cylinder to which a 0.2 mm stainless steel plate with a 2 mm × 4 mm slotted hole is attached. For specimen mounting, a drop of agarose (5–7 µl) was pipetted onto the center of the slotted hole and excessive liquid was removed to create an agarose film with a thickness of around 50 µm. Using a small paint brush, dechorionated medfly embryos were placed onto the agarose film with their elongated anterior-posterior axis aligned with the long axis of the slotted hole (Fig. 1b). The embryos were only partially embedded in agarose, thus keeping the distance that the laser beam and the emitted fluorescence must pass through agarose at a minimum while facilitating the necessary gas exchange with the imaging buffer.

Upon insertion into the sample chamber, the cobweb holder was aligned along *x* (Fig. 1c, first column), which we define as the preliminary direction (orientation −45°) to simplify translation of the embryo into the center of the field of view. For imaging, the cobweb holder was rotated by 45°, which we define as direction 1 (orientation 0°). Along this direction, the steel plate blocked neither the illumination, detection or transmission light (Fig. 1c, second and third column), which permitted to record the embryos in both, the transmission light (Fig. 1d) and fluorescence (Fig. 1e) channels. The cobweb holder was rotated around *y* in steps of 90° to record the specimen along four directions (Supplementary Fig. 1). The extents of the cobweb holder allowed the acquisition of *z* stacks with a range of up to 800 µm. Post-acquisition corrections such as drift compensation were not necessary.

### Mounting using the agarose hemisphere method

In LSFM, the lateral resolution (along *x* and *y*) exceeds the axial resolution (along *z*) by a factor of approximately three to four^53^. Optical sectioning of insect embryos using the cobweb holder is restricted to planes along the ventro-dorsal and lateral axes. To also achieve optical sectioning along the antero-posterior axis that benefit from the high lateral resolution, we adapted the agarose hemisphere mounting method, which was initially established for *T. castaneum* embryos^17,18^. However, unlike in the initial approach, medfly embryos were attached with their dorsal side to the pole of the agarose hemisphere (Supplementary Fig. 2).

### Long-term live imaging and embryo retrieval

In total, nine long-term live imaging datasets (DS) were recorded (Supplementary Table 1). In six out of nine datasets (DS0001–DS0006), embryos were captured *in toto* under equal conditions for about 60 h with a temporal interval of 30 min, covering approximately 97% of embryonic development (Supplementary Video 1). The remaining datasets cover specific embryonic processes, e.g., pole cell formation (DS0007) and head involution (DS0008 and DS0009). For datasets DS0001–DS0008, embryos were imaged along four directions along the orientations 0°, 90°, 180° and 270°. Embryos from DS0001 and DS0003 were imaged along both ventrolateral-dorsolateral axes, whereas embryos from datasets DS0002, DS0004, and DS0005 were imaged along the ventro-dorsal and lateral axes. For dataset DS0009, the embryo was imaged along two directions in the orientations 0° and 90°. For each acquired plane, the embryo was illuminated with a 488 nm laser beam with a power of 135 µW during a 50 ms exposure time window. Each *z* stack consisted of 100 or 115 planes with an axial pitch of 2.58 µm. Thus, the datasets were acquired with a *x*:*y*:*z* resolution ratio of 1:1:4 and 1:1:8 for the 10× objective and the 20× objective, respectively. After imaging was completed, embryos were retrieved from the microscope. All embryos hatched morphologically intact. However, the embryo from DS0006 did not develop into a healthy adult, hence the respective image data was excluded from further analysis.

### Image processing

The *z* stacks and respective *z* maximum projections were rotated around *z* and cropped to a final size of 500 × 1390 pixel to align the anterior-posterior axis of the embryos with the *y* axis of the image and place the embryo in the center of the *z* stacks and *z* maximum projections. For each direction, all *z* maximum projections were combined, and all time points were subsequently concatenated to *t* stacks, which were subjected to a mean transformation as described previously^18^ and adjusted in brightness and contrast.

### Two-level staging system implementation

**Fig. 2:**
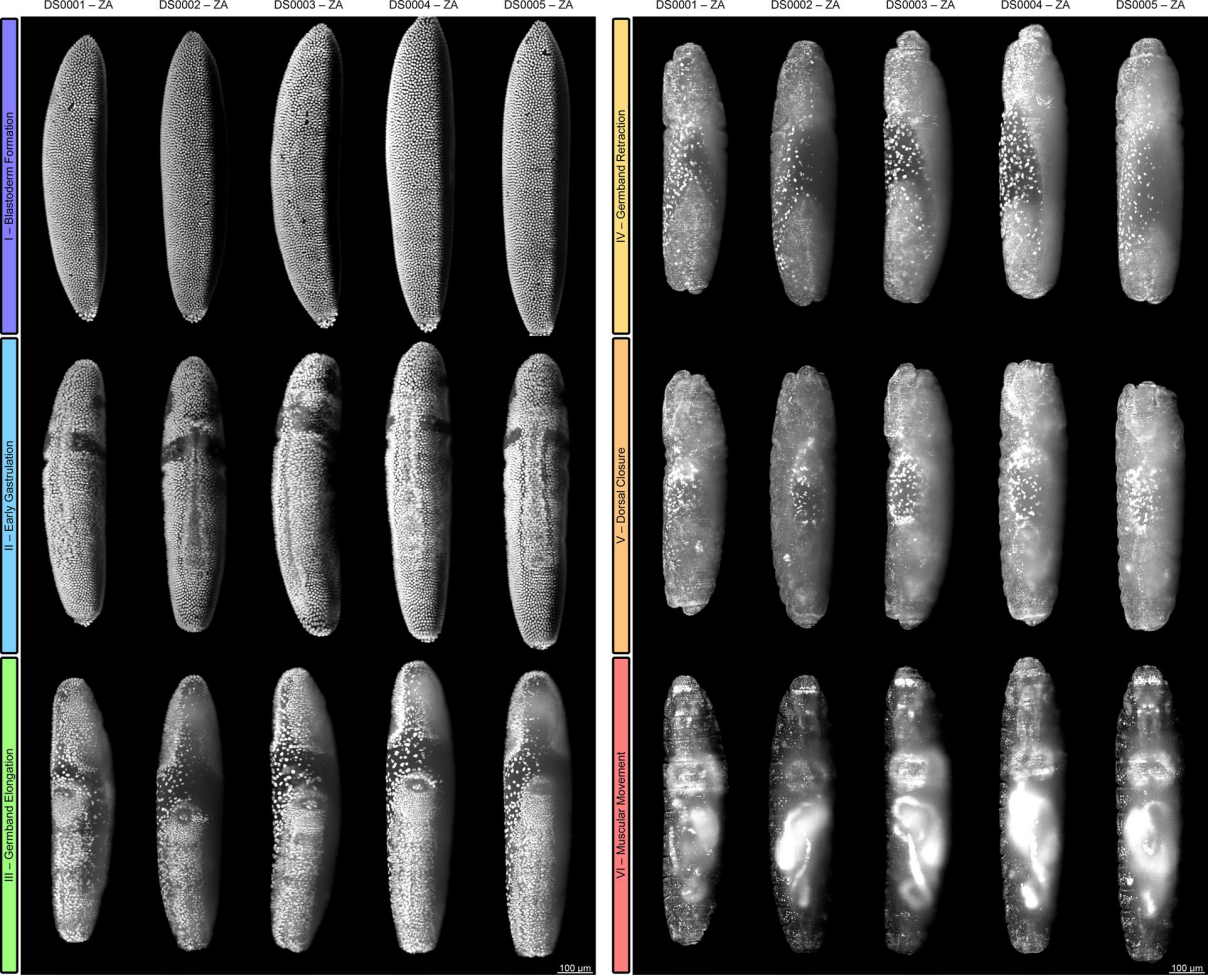
Comparison of datasets DS0001–DS0005 for the embryogenetic events I – blastoderm formation, II – early gastrulation, III – germband elongation, IV – germband retraction, V – dorsal closure, and VI – muscular movement. For this overview, time points were chosen that show characteristic structures for the respective event. DS0001 and DS0003 are depicted ventrolateral during I and II, and dorsolateral during III to VI, whereas DS0002, DS0004 and DS0005 are depicted ventral during I and II and dorsal during III to VI. Embryogenetic events are color-coded (see Methods section). ZA, *z* maximum projection with image adjustment.

For proper temporal quantification of embryonic development and to provide a standard for future studies of the medfly, a comprehensive staging system is necessary. The proposed staging system consists of two levels, *i.e*., embryogenetic events and stages, relies on five *in toto* datasets (DS0001–DS0005) that were acquired under equal conditions, and considers exclusively morphogenetic criteria. For the upper level, six consecutive embryogenetic events are specified and denoted with color-coded Roman numerals: (I) blastoderm formation is represented in blue, (II) early gastrulation in cyan, (III) germband elongation in green, (IV) germband retraction in yellow, (V) dorsal closure in orange and (VI) muscular movement in red. A comparison of event-characteristic structures between DS0001–DS0005 is given in Fig. 2. A comprehensive overview of embryonic event onset time points is shown Supplementary Fig. 3 and summarized in Supplementary Table 2. Since imaging started approximately 2 h after embryo collection, the first time point (TP0001) was set to 02:00 h of absolute development time.

For the lower level, due to the high similarity, the 17 stages from the *D. melanogaster* staging system^54^ were adapted analog to what has been done in a similar study on *M. abdita*^38^ and denoted with Arabic numerals. In consequence, each embryogenetic event consists of one or multiple stages. Major event and stage identifiers are given in Table 1. A comprehensive overview of stage onset time points for DS0001–DS0005 along one orientation is shown in Supplementary Fig. 4 and summarized in Supplementary Table 3.

**Table 1:** Alignment and temporal breakdown of datasets DS0001–DS0005 into embryogenetic events (Roman numbers) and stages (Arabic numbers).

Event/Stage	Identifier	DS0001 (median)			DS0002 (longer)			DS0003 (longer)			DS0004 (shorter)			DS0005 (shorter)			Standard Deviations	
		TP	time	rel dev	TP	time	rel dev	TP	time	rel dev	TP	time	rel dev	TP	time	rel dev	time	rel dev
I-1 Blastoderm Formation	egg fertilization results in the zygote, which consists of the yolk and the zygotic nuclei	—	00:00 h	0.00%	—	00:00 h	0.00%	—	00:00 h	0.00%	—	00:00 h	0.00%	—	00:00 h	0.00%	± 00:00 h	± 0.0%
I-2	the yolk begins withdrawing from both poles	1	02:00 h	3.20%	1	02:00 h	3.10%	1	02:00 h	3.20%	1	02:00 h	3.30%	1	02:00 h	3.30%	± 00:00 h	± 0.1%
I-3	the yolk withdrawal at both poles reaches a relative maximum and starts reversing	4	03:30 h	5.60%	4	03:30 h	5.40%	4	03:30 h	5.60%	4	03:30 h	5.80%	4	03:30 h	5.70%	± 00:00 h	± 0.2%
I-4	the last zygotic nuclei reach the surface	6	04:30 h	7.20%	7	05:00 h	7.70%	6	04:30 h	7.10%	6	04:30 h	7.50%	6	04:30 h	7.40%	± 00:13 h	± 0.2%
I-5	the blastoderm nuclei complete the 13^th^ synchronous nuclear division	10	06:30 h	10.40%	11	07:00 h	10.80%	10	06:30 h	10.30%	10	06:30 h	10.80%	10	06:30 h	10.70%	± 00:13 h	± 0.2%
II-6 Early Gastrulation	the blastoderm nuclei start rearranging after an extended period of quiescence	20	11:30 h	18.40%	22	12:30 h	19.20%	21	12:00 h	19.00%	19	11:00 h	18.30%	19	11:00 h	18.00%	± 00:39 h	± 0.5%
II-7	the anterior midgut primordium emerges and invaginates at the anterior tip of the ventral furrow	24	13:30 h	21.60%	26	14:30 h	22.30%	25	14:00 h	22.20%	23	13:00 h	21.70%	23	13:00 h	21.30%	± 00:39 h	± 0.5%
III-8 Germband Elongation	the posterior tip of the germband begins anteriad elongation along the dorsal side towards with high speed	26	14:30 h	23.20%	27	15:00 h	23.10%	26	14:30 h	23.00%	24	13:30 h	22.50%	24	13:30 h	22.10%	± 00:40 h	± 0.5%
III-9	the posterior tip of the germband continues anteriad elongation along the dorsal side with moderate speed	30	16:30 h	26.40%	31	17:00 h	26.20%	30	16:30 h	26.20%	29	16:00 h	26.70%	29	16:00 h	26.20%	± 00:25 h	± 0.2%
III-10	the posterior tip of the germband continues anteriad elongation along the dorsal side with low speed	33	18:00 h	28.80%	32	17:30 h	26.90%	32	17:30 h	27.80%	31	17:00 h	28.30%	31	17:00 h	27.90%	± 00:25 h	± 0.7%
III-11	the posterior tip of the germband completes anteriad elongation and remains in a quiescent position	41	22:00 h	35.20%	43	23:00 h	35.40%	38	20:30 h	32.50%	36	19:30 h	32.50%	38	20:30 h	33.60%	± 01:23 h	± 1.4%
IV-12 Germband Retraction	the posterior tip of the germband begins posteriad retraction along the dorsal side	46	24:30 h	39.20%	48	25:30 h	39.20%	46	24:30 h	38.90%	44	23:30 h	39.20%	44	23:30 h	38.50%	± 00:50 h	± 0.3%
V-13 Dorsal Closure	the posterior tip of the germband completes posteriad retracting	61	32:00 h	51.20%	62	32:30 h	50.00%	59	31:00 h	49.20%	53	28:00 h	46.70%	55	29:00 h	47.50%	± 01:56 h	± 1.8%
V-14	the clypeolabrum turns from an antero-dorsal to an antero-ventral orientation	66	34:30 h	55.20%	68	35:30 h	54.60%	66	34:30 h	54.80%	62	32:30 h	54.20%	62	32:30 h	53.30%	± 01:20 h	± 0.7%
V-15	the abdomen withdrawal at the posterior pole reaches the absolute maximum	73	38:00 h	60.80%	74	38:30 h	59.20%	71	37:00 h	58.70%	69	36:00 h	60.00%	71	37:00 h	60.70%	± 00:58 h	± 0.9%
VI-16 Muscular Movement	the dorsal epidermal primordia complete medial fusion and turn into the dorsal epidermis	87	45:00 h	72.00%	84	43:30 h	66.90%	86	44:30 h	70.60%	79	41:00 h	68.30%	82	42:30 h	69.70%	± 01:36 h	± 2.0%
VI-17	the posterior tip of the ventral cord shortens to the 5^th^ segment of the abdomen	106	54:30 h	87.20%	110	56:30 h	86.90%	104	53:30 h	84.90%	97	50:00 h	83.30%	101	52:00 h	85.20%	± 02:27 h	± 1.6%
Hatch	the embryo completes embryonic development, hatches and turns into the larva	122	62:30 h	100.00%	127	65:00 h	100.00%	123	63:00 h	100.00%	117	60:00 h	100.00%	116	59:30 h	100.00%	± 02:15 h	± 0.0%

Onset time points of embryogenetic events are color-coded. Imaging typically begins with stage I-2, the values for I-1 are extrapolated (see Methods section). TP, imaging time point; time, absolute time passed from the onset of I-1 until the indicated TP; rel dev, relative progress of embryonic development from the onset of I-1 until the indicated TP.

### Dataset alignment

Of the five datasets (DS0001–DS0005) in which the embryos were imaged *in toto* and under identical conditions, DS0001 was the dataset with the median development time, so DS0002–DS0005 were adjusted and aligned stage-by-stage onto DS0001. In cases where stages in DS0002–DS0005 lasted shorter or longer, the first and last time points were aligned to DS0001, resulting in stretching or compression of intermediate periods, respectively. As this leads to non-matching time points, the respective matching time points were interpolated. The interpolation values were used for calculations of the absolute and relative standard deviations (Supplementary Table 4). An overview of embryogenetic events and stages for DS0001–DS0005 is given in Table 1. A comprehensive overview of stage onset time points for DS0001–DS0005 is shown in Supplementary Table 4.

## Data Records

The nine long-term datasets of medfly embryonic development are provided as ZIP-compressed TIFF files and explore six degrees of freedom: the first (*x*) and second (*y*) spatial dimensions are obtained simultaneously during one camera exposure period. The third spatial dimension (*z*) is represented by the optical sections that are recorded while the embryo is moved through the light sheet. The *z* stacks are saved as individual files using the TIFF-intrinsic container function (indicated as PL(ZS) within the file name). Together, the three spatial dimensions define the volume of view. The further degrees of freedom are the fluorescence channel (one), the direction (typically four) and the time point (up to 126), which are saved individually (indicated as CH, DR or TP within the subfolder or file name, respectively). For convenience, *z* maximum projections are also provided. These are simplifications of the datasets where one spatial dimension (*z*) is removed. The projections are provided in two versions, as raw *z* maximum projections (indicated as PL(ZM) within the subfolder or file name) or as *z* maximum projections with image adjustment (indicated as PL(ZA) within the subfolder or file name). Respective *t* stacks are saved as individual files using the TIFF-intrinsic container function (indicated as TP(TS) within the file name), the adjusted versions are further provided as direction montages along *x* and *y* (indicated as DR(AX) or (AY) within the file name). For each dataset, all files were compiled into one ZIP folder and deposited as a single record at Zenodo^55–63^. In addition to the ZIP folder, each record also contains a downscaled AVI movie of the direction montage along *x* (12–60 Mb) for fast inspection as well as a machine-readable XLSX metadata file. Metadata optimized for human readability and DOI-based access information are provided in Supplementary Table 1.

## Technical Validation

### Microscope calibration

Prior to each imaging assay, the DSLM went through a two-step calibration routine as described previously^18^. Thus, typical problems that might occur in LSFM are avoided (*e.g*., offset or tilt between the light sheet and the focal plane of the detection objective). Laser power (Supplementary Table 1) was measured with an optical power and wavelength meter (OMM-6810B and OMH-6703B, Newport, Irvine, CA, United States) at the exact location where the embryos were positioned during the imaging process.

### Quality control

Imaged embryos were raised to adults to assure that the imaging procedure, *e.g*., the irradiance by the laser, does not induce any aberrations. The embryos were imaged until hatching, *i.e*., until the first time point in which only the empty eggshell was captured. When this happened, imaging was stopped and the larva, which was floating in the imaging buffer of the sample chamber, was retrieved with a plastic Pasteur pipet. The larva was placed on larval medium^64^ and incubated for several weeks under the same conditions as described for the adult culture in the Methods section until pupation and eclosure occurred. The embryo from dataset DS0006 did not develop into a healthy adult and was therefore excluded from further analysis. The quality of the image data was validated by manual examination of all *z* maximum projections: for each dataset (nine in total), for each time point (up to 126 per dataset), and for each direction (two to four per dataset and time point).

### Stage onset identifiers

Iconic aspects for almost all stage onsets defined for *Drosophila melanogaster*^54^ could also be identified in *Ceratitis capitata*. The only deviation concerned the transition from stage I-5 to stage II-6 for technical reasons. For the fruit fly, one of the central morphogenetic processes is the completion of cellularization, which cannot be recognized in the presented datasets as the fluorescent medfly line provides only signals in the nuclei. Therefore, the identifier for the onset of stage II-6 was defined as the first noticeable movement of the blastoderm nuclei after the 13^th^ synchronous nuclear division.

### Temporal resolution

The datasets used to establish the staging system, *i.e*., DS0001–DS0005, cover, on average, 120 time points. In conjunction with the extrapolation for stage I-1, which equals four time points for each dataset (Supplementary Table 1), this corresponds to a relative temporal resolution of approximately 0.8% of embryonic development. Consequently, stage onset time points may deviate by up to 0.4%, which may be particularly noticeable if a developmental period features extensive morphogenetic changes, for example stage III-8. In the images that were specified as the stage onset time points, the embryos from DS0001 and DS0003 appear to be slightly ahead in development compared the embryos from the remaining three datasets considering the position of the posterior germband tip (Supplementary Table 3d, first row).

At an imaging temperature of 23 °C, the average development time of embryos from DS0001–DS0005 was 59:30 ± 2:15 h (Supplementary Table 4). These values compare relatively accurately to previous measurements of embryonic development time at 20 °C and 25 °C^51^.

## Usage Notes

To work with the data, ImageJ^65,66^ or its derivate FIJI^67^ are recommended as the primary image processing program, which are open-source software frameworks for the analysis of multi-dimensional biological imaging data. The order of the degrees of freedom (see Data Records section) is compatible with all image processing approaches capable of handling three-dimensional, multi-channel, multi-view dynamic data.

## Supplementary information

Supplementary Material

Supplementary Video 1

## Data Availability

The custom *Mathematica* (Wolfram Research, version 13.0) script used to subject the *t* stacks to a mean transformation has been described previously^18^ (this function does not require any parameters).
