# Supplementary material for: In toto light sheet fluorescence microscopy live imaging datasets of Ceratitis capitata embryonic development
Source: Sci Data. 2022 Jun 15;9:340. doi: 10.1038/s41597-022-01443-x (PMC9200851; doi:10.1038/s41597-022-01443-x)
Supplement: Supplementary file 1 — Supplementary Material [file 41597_2022_1443_MOESM1_ESM.pdf]

Title

In toto light sheet fluorescence microscopy live imaging datasets of *Ceratitis capitata* embryonic development

Authors

Frederic Strobl[1], Marc F. Schetelig[2] and Ernst H.K. Stelzer[1]

Affiliations

[1]Physical Biology / Physikalische Biologie (IZN, FB 15)

Buchmann Institute for Molecular Life Sciences (BMLS)

Cluster of Excellence Frankfurt – Macromolecular Complexes (CEF – MC)

Goethe-Universität – Frankfurt am Main (Campus Riedberg)

Max-von-Laue-Straße 15 – D-60438 Frankfurt am Main – Germany

[2]Justus-Liebig-Universität Gießen

Department of Insect Biotechnology in Plant Protection

Winchesterstraße 2 – D-35394 Gießen – Germany

Corresponding authors:

Frederic Strobl (frederic.strobl@physikalischebiologie.de)

Ernst H.K. Stelzer (ernst.stelzer@physikalischebiologie.de)

Table of Contents

Supplementary Figures

- Supplementary Figure 1
- Supplementary Figure 2
- Supplementary Figure 3
- Supplementary Figure 4

Supplementary Tables

- Supplementary Table 1
- Supplementary Table 2
- Supplementary Table 3
- Supplementary Table 4

Supplementary Videos

- Supplementary Video 1 Caption

Supplementary Figures

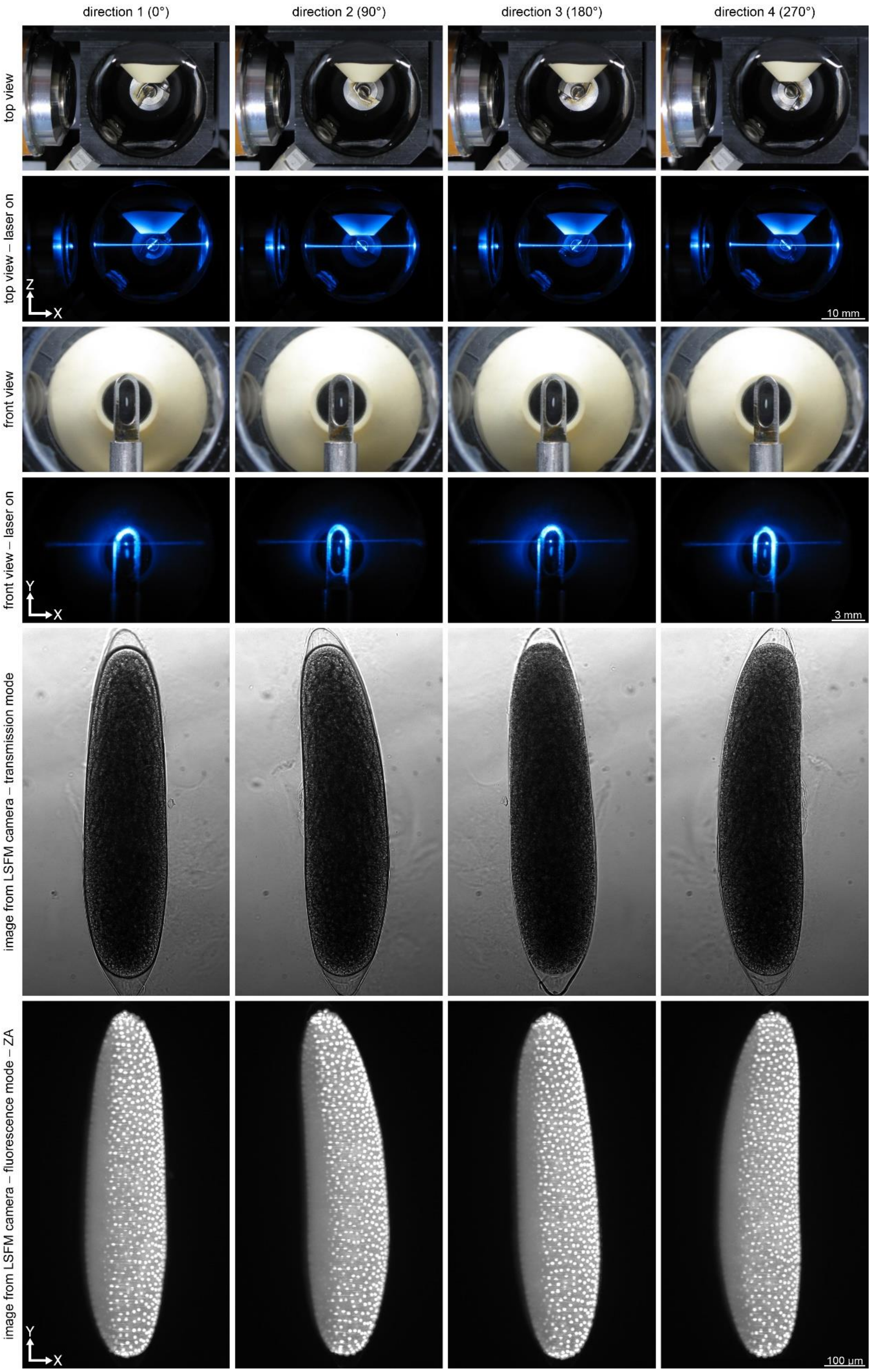

Supplementary Figure 1 – Illustration of the medfly embryo imaging process with the cobweb holder along four directions. Top view (first and second row) and front view (third and fourth row) of the sample chamber illustrating the illumination process along each direction. Medfly embryo imaged during blastoderm formation in the transmission light (fifth row) and fluorescence (sixth row) channels along four directions (orientations 0°, 90°, 180°, 270°). ZA, *z* maximum projection with image adjustment.

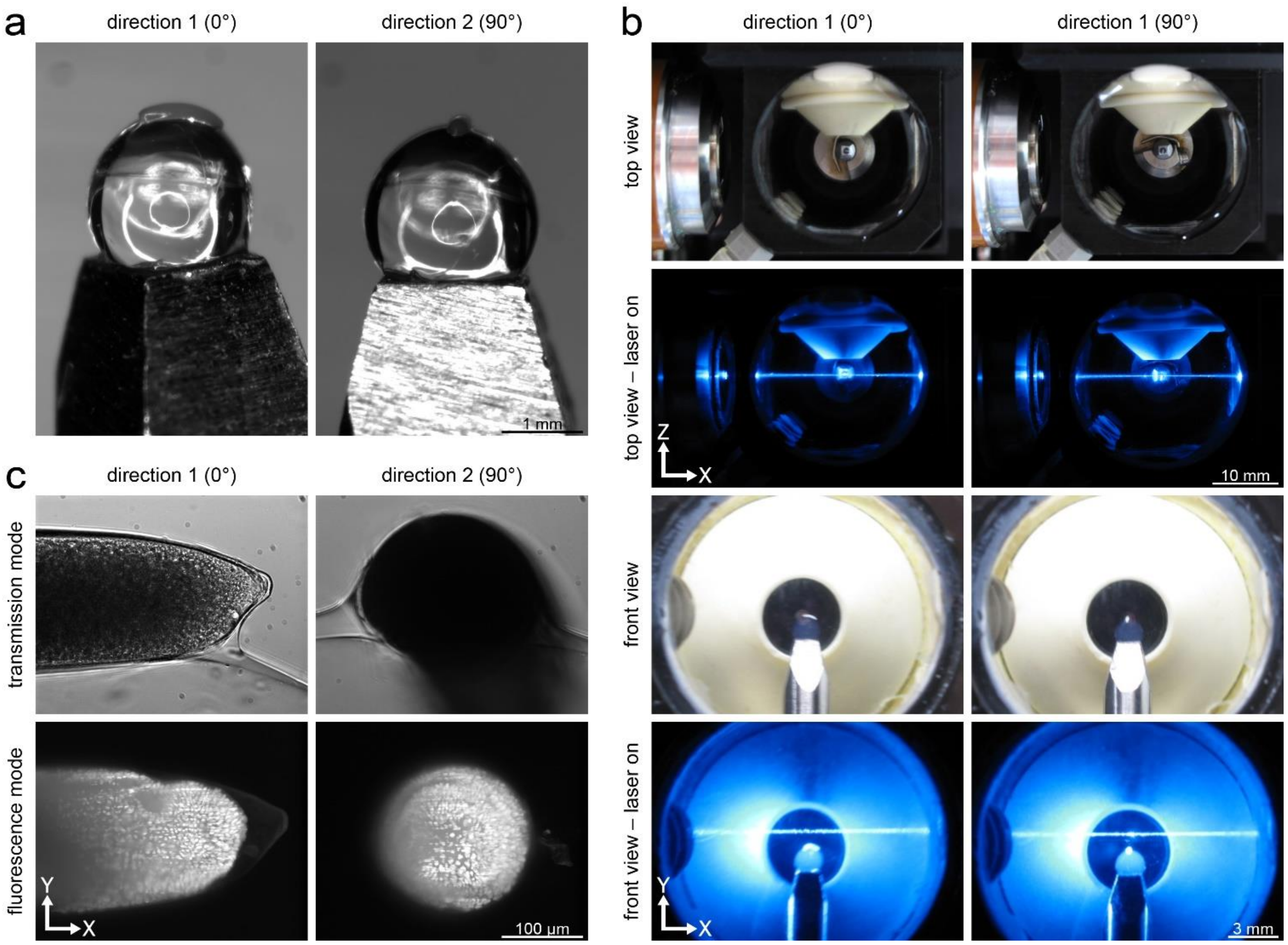

Supplementary Figure 2 – Illustration of medfly embryo imaging process using the agarose hemisphere mounting method. (a) Front and side view of the sample holder with embryo placed on top of the agarose hemisphere. The shown views correspond to the orientations 0° and 90°. **(b)** Top view (first and second row) and front view (third and fourth row) of the sample chamber using the alternative mounting method. **(c)** Medfly embryo imaged in the transmission light (upper row) and fluorescence channel (lower row) along two directions (orientations 0° and 90°).

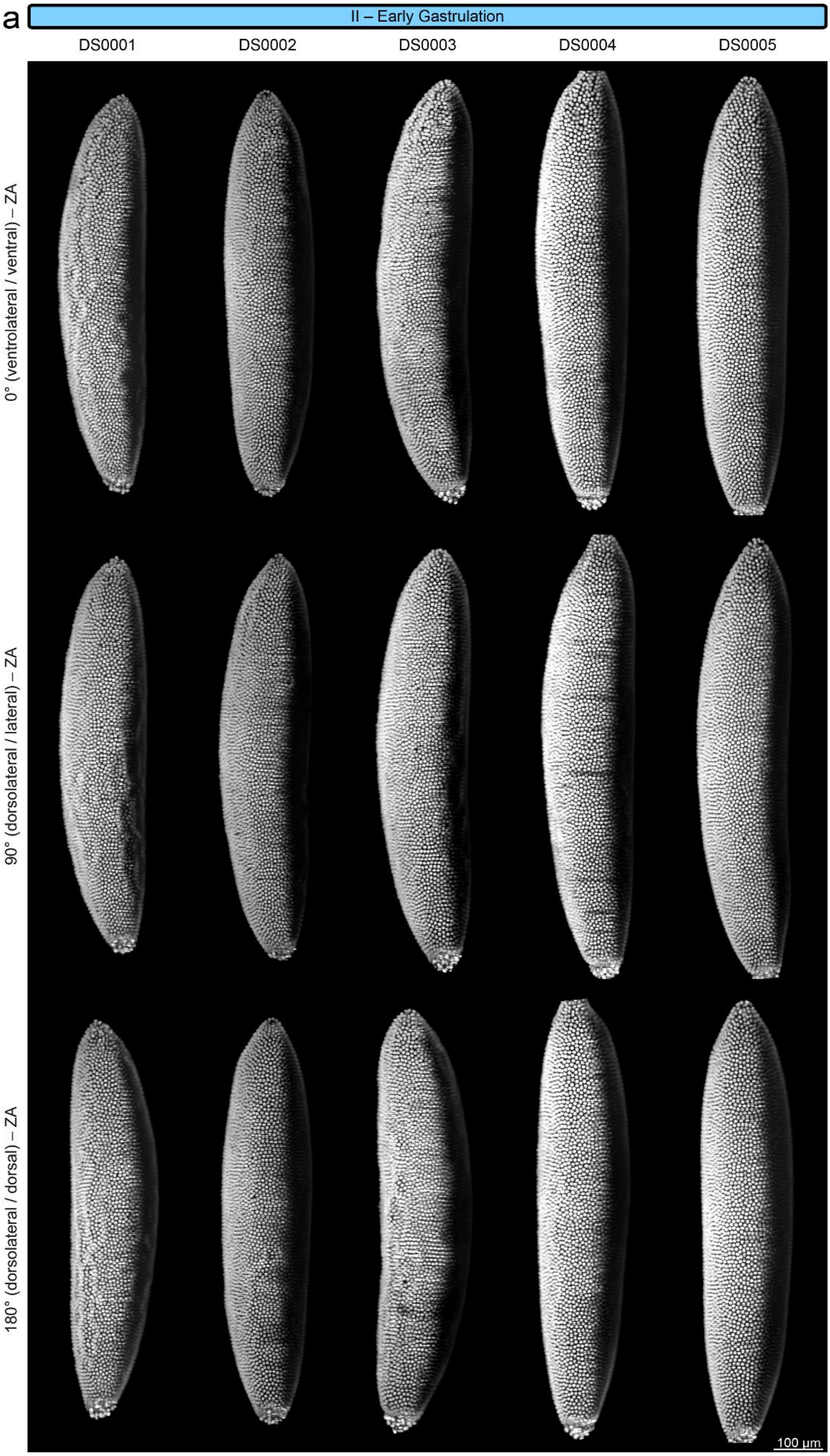
a
II – Early Gastrulation
DS0001
DS0002
DS0003
DS0004
DS0005
0° (ventrolateral / ventral) – ZA
90° (dorsolateral / lateral) – ZA
180° (dorsolateral / dorsal) – ZA
100 µm

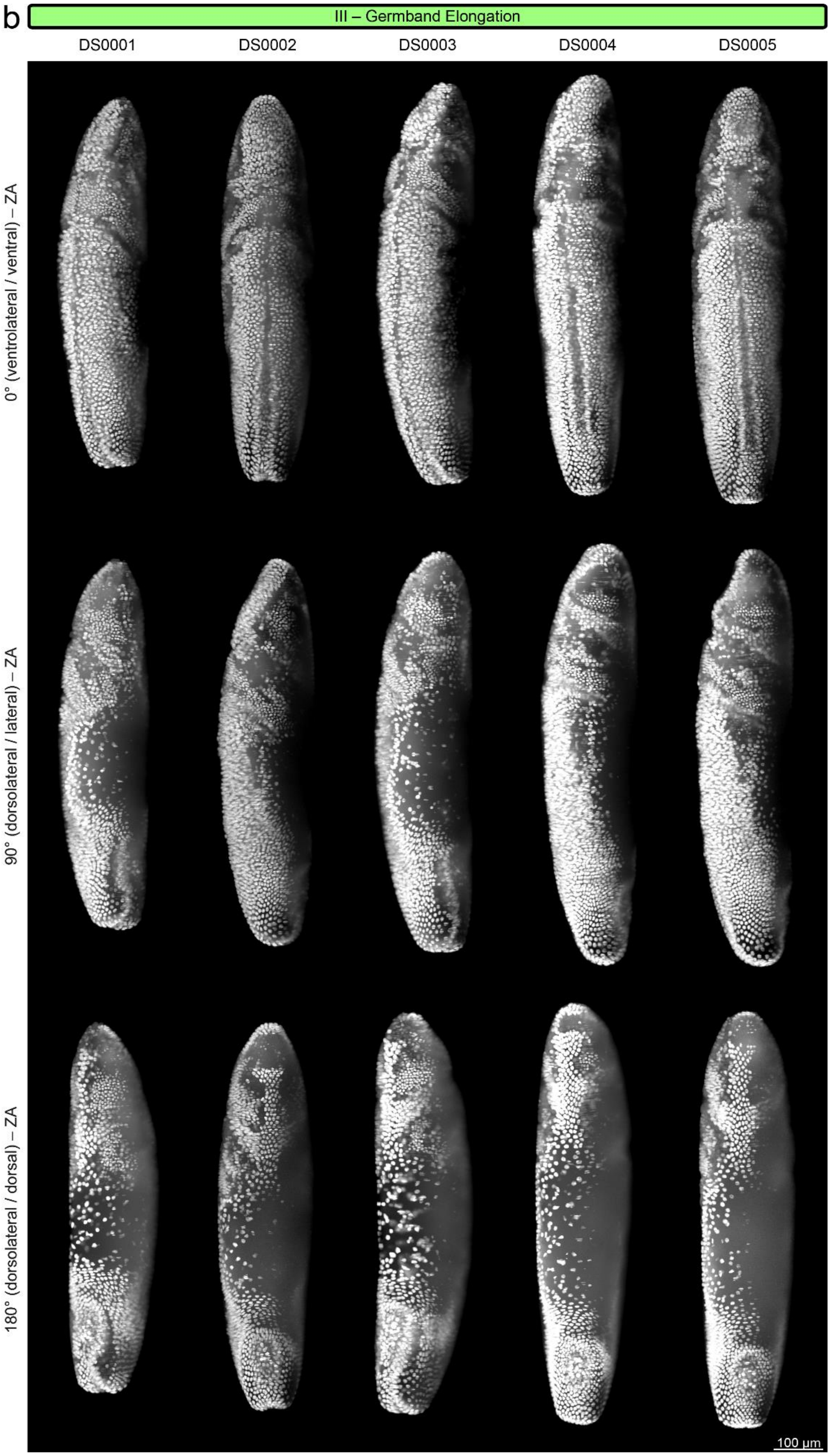
b
III – Germband Elongation
DS0001
DS0002
DS0003
DS0004
DS0005
0° (ventrolateral / ventral) – ZA
90° (dorsolateral / lateral) – ZA
180° (dorsolateral / dorsal) – ZA
100 µm

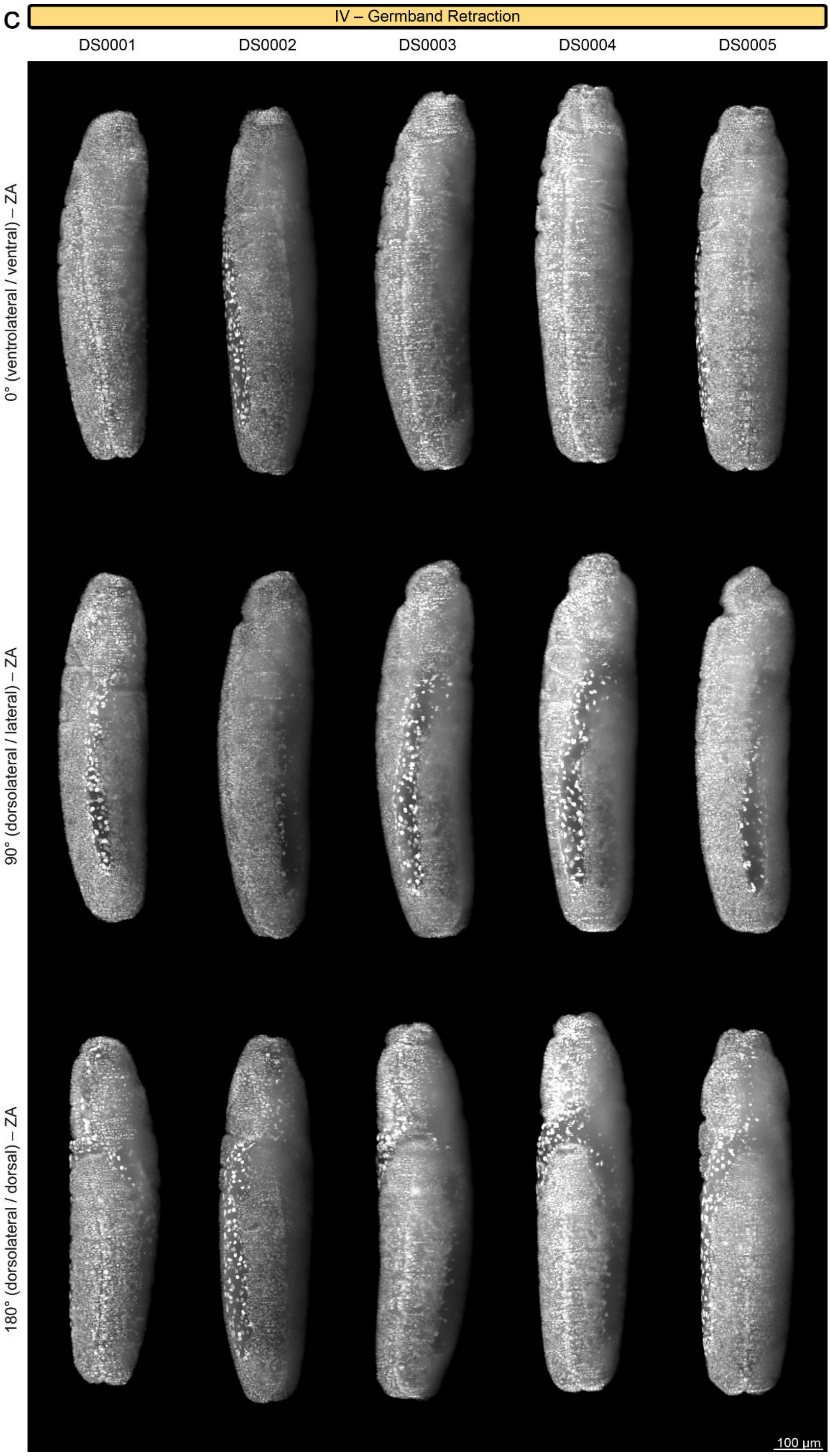
C
IV – Germband Retraction
DS0001
DS0002
DS0003
DS0004
DS0005
0° (ventrolateral / ventral) – ZA
90° (dorsolateral / lateral) – ZA
180° (dorsolateral / dorsal) – ZA
100 µm

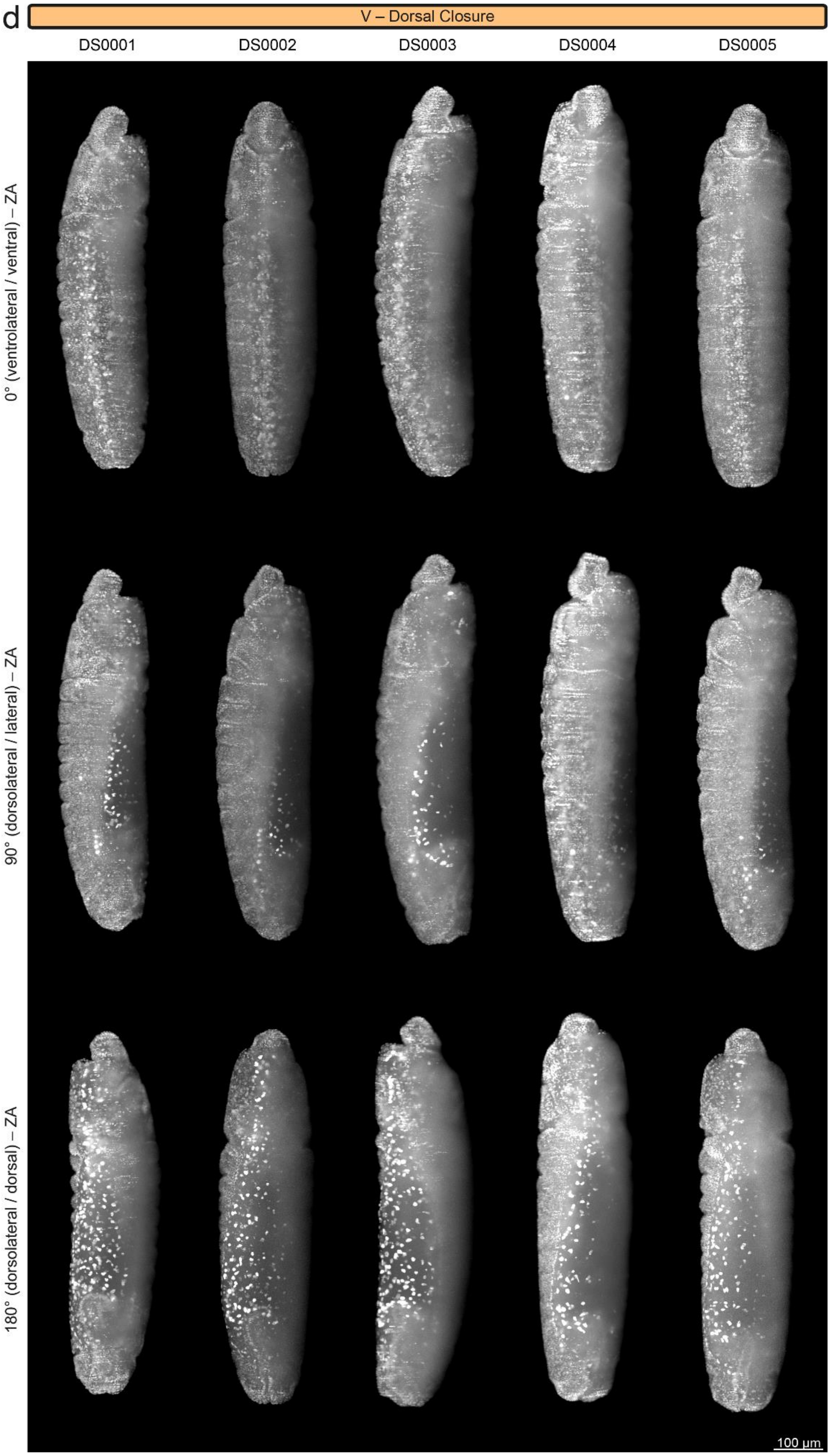
d
V – Dorsal Closure
DS0001
DS0002
DS0003
DS0004
DS0005
0° (ventrolateral / ventral) – ZA
90° (dorsolateral / lateral) – ZA
180° (dorsolateral / dorsal) – ZA
100 µm

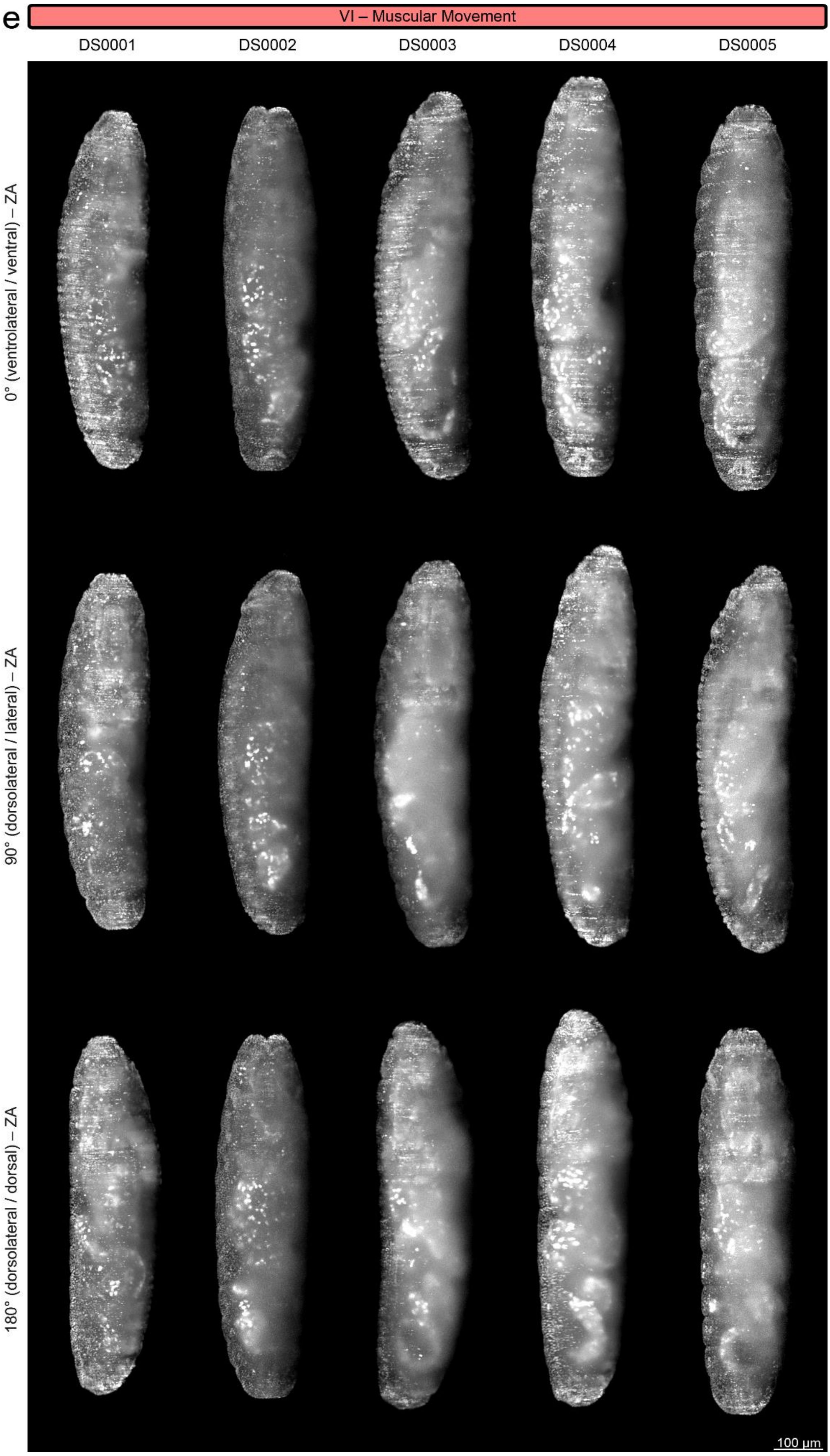

Supplementary Figure 3 – Comparison of datasets DS0001–DS0005 at the onset of the embryogenetic events (a) early gastrulation, (b) germband elongation, (c) germband retraction, (d) dorsal closure and (e) muscular movement along three directions (orientations 0°, 90° and 180°). Since recording started after the onset of blastoderm formation, respective image data is not available. Embryos from datasets DS0001 and DS0003 are depicted ventrolateral, dorsolateral and dorsolateral, whereas embryos from DS0002, DS0004 and DS0005 are depicted ventral, lateral and dorsal. ZA, Z maximum projection with image adjustment.

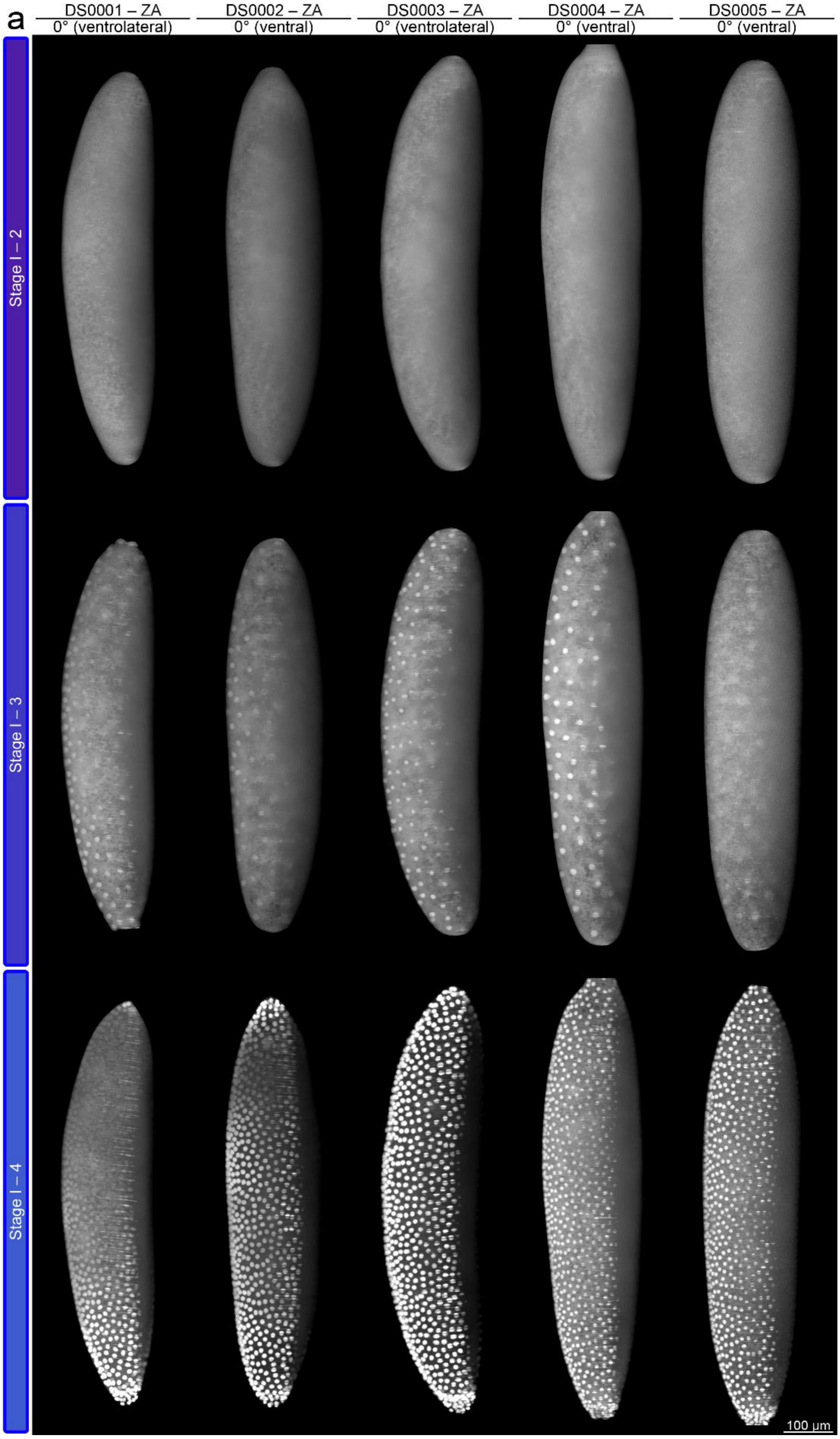
a
DS0001 – ZA
0° (ventrolateral)
DS0002 – ZA
0° (ventral)
DS0003 – ZA
0° (ventrolateral)
DS0004 – ZA
0° (ventral)
DS0005 – ZA
0° (ventral)
Stage I – 2
Stage I – 3
Stage I – 4
100 µm

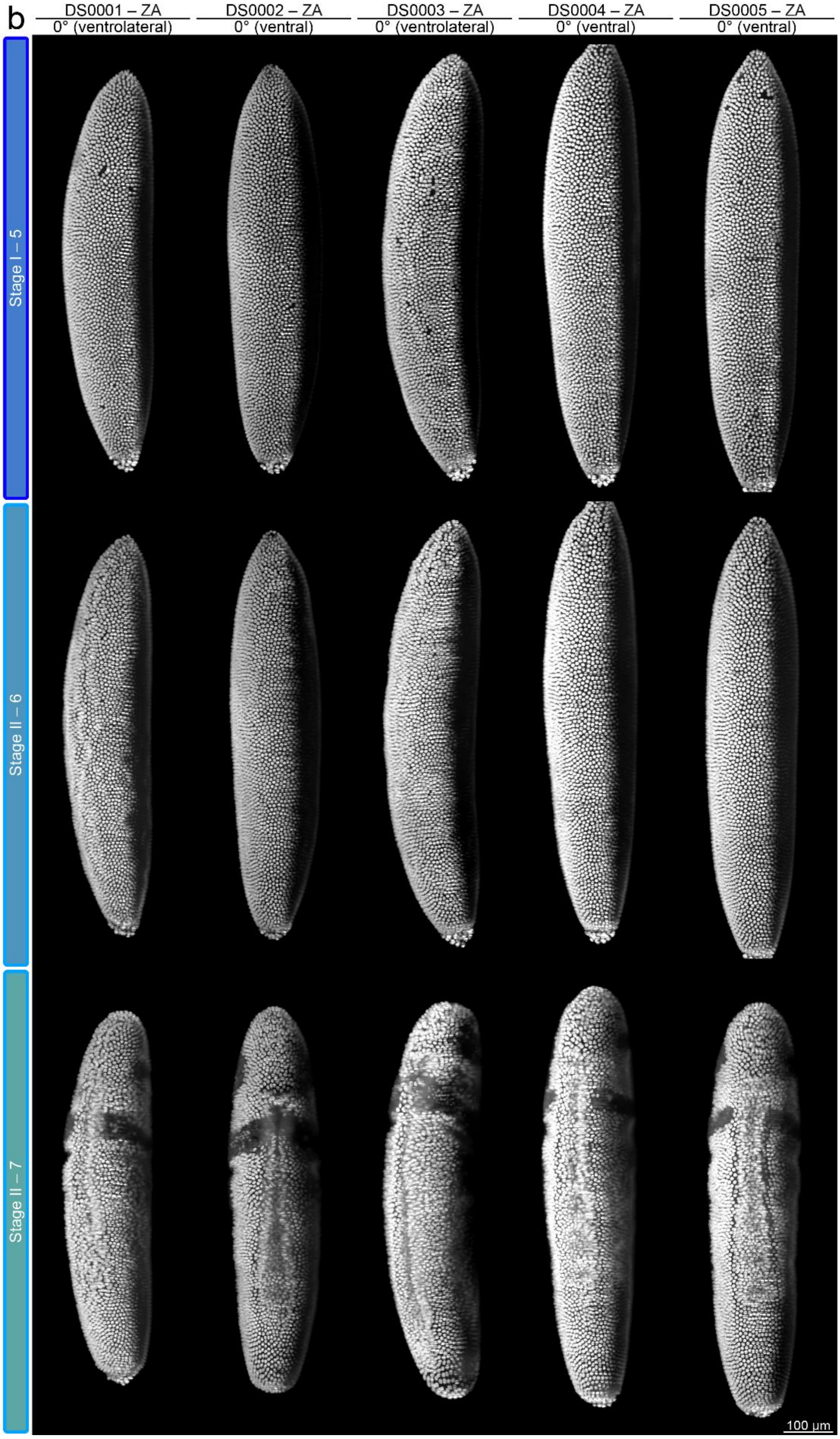
b
DS0001 – ZA
0° (ventrolateral)
DS0002 – ZA
0° (ventral)
DS0003 – ZA
0° (ventrolateral)
DS0004 – ZA
0° (ventral)
DS0005 – ZA
0° (ventral)
Stage I – 5
Stage II – 6
Stage II – 7
100 µm

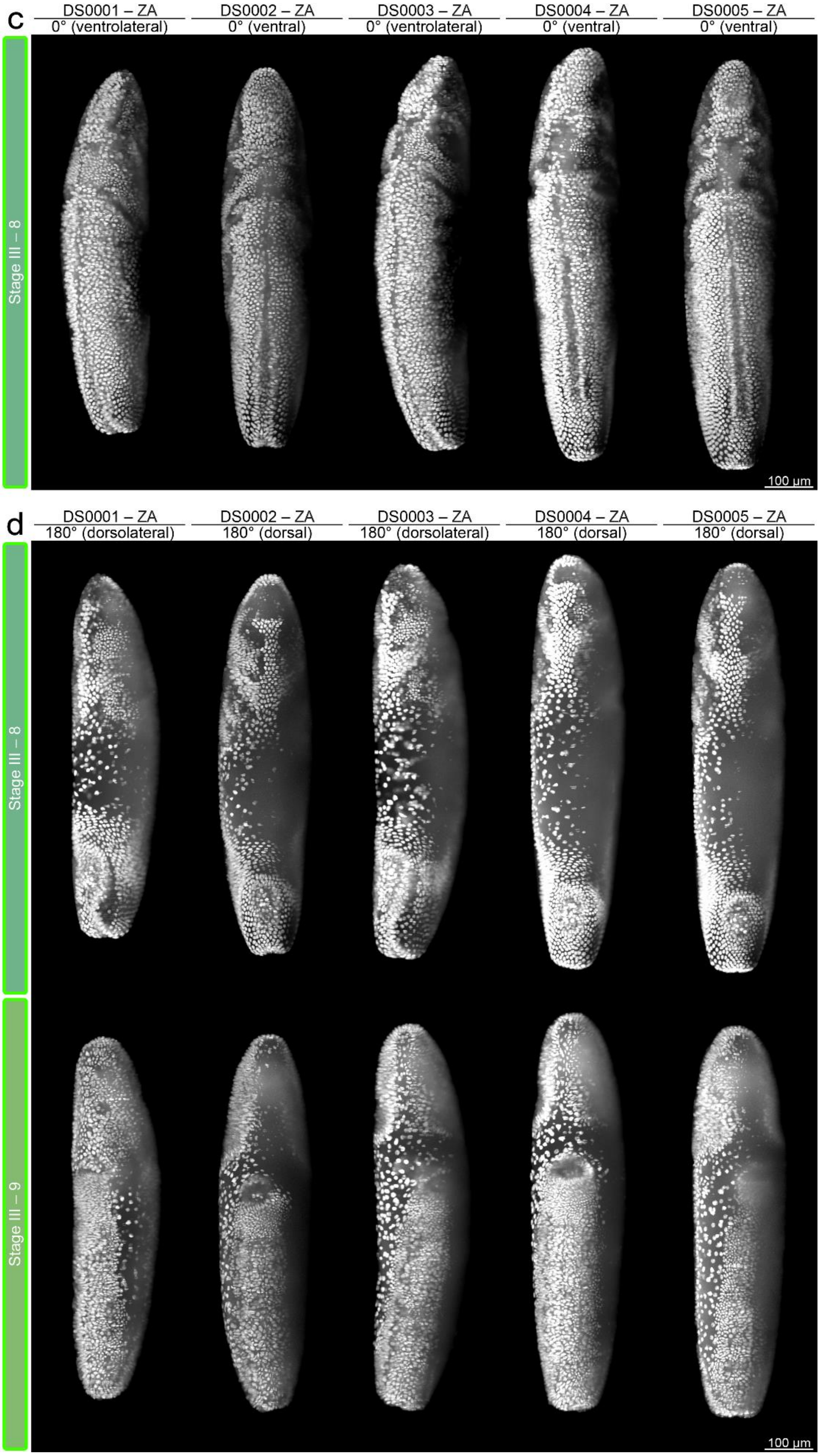
c
DS0001 – ZA
0° (ventrolateral)
DS0002 – ZA
0° (ventral)
DS0003 – ZA
0° (ventrolateral)
DS0004 – ZA
0° (ventral)
DS0005 – ZA
0° (ventral)
Stage III – 8
100 µm
d
DS0001 – ZA
180° (dorsolateral)
DS0002 – ZA
180° (dorsal)
DS0003 – ZA
180° (dorsolateral)
DS0004 – ZA
180° (dorsal)
DS0005 – ZA
180° (dorsal)
Stage III – 8
Stage III – 9
100 µm

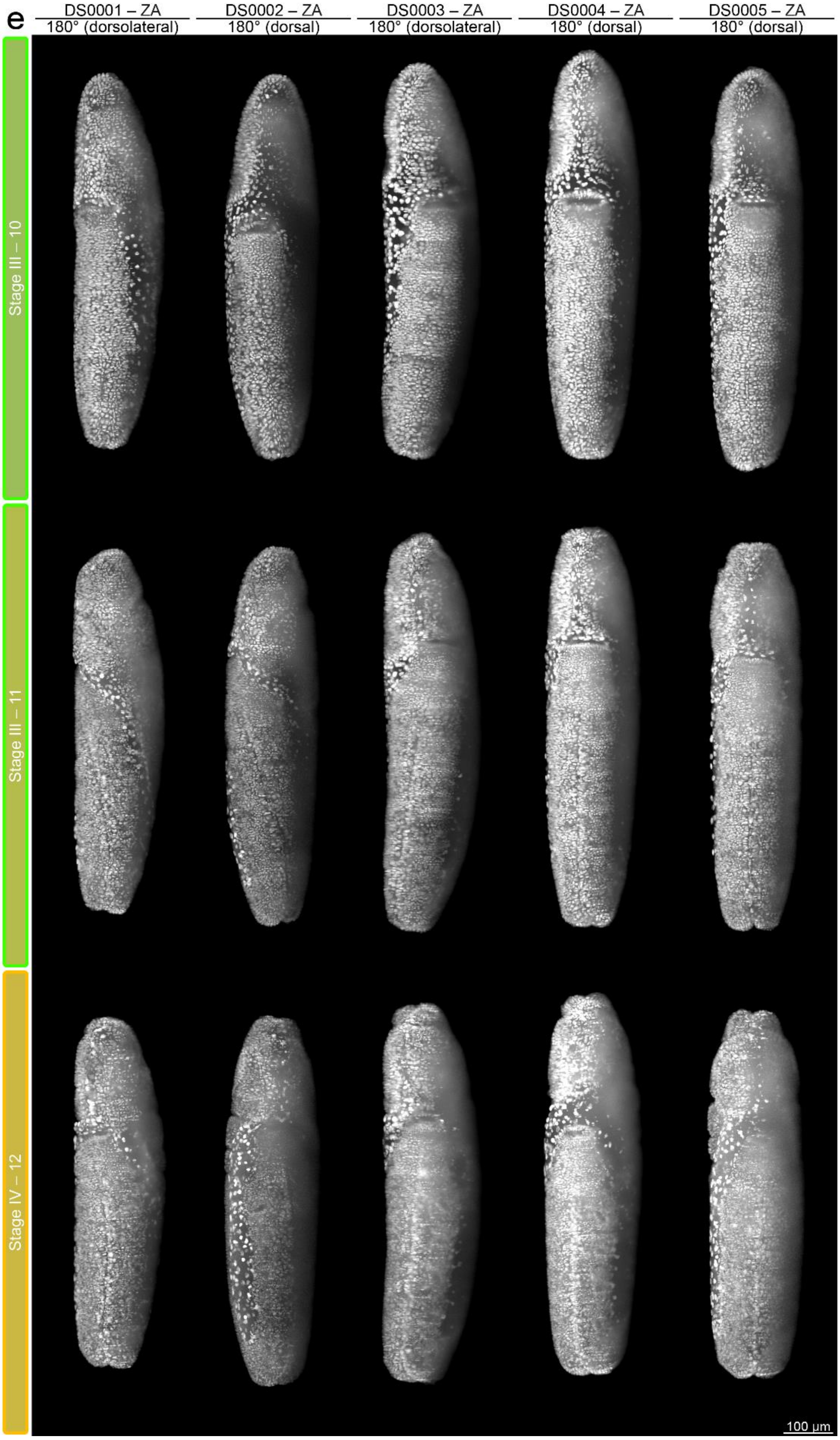
e
DS0001 – ZA
180° (dorsolateral)
DS0002 – ZA
180° (dorsal)
DS0003 – ZA
180° (dorsolateral)
DS0004 – ZA
180° (dorsal)
DS0005 – ZA
180° (dorsal)
Stage III – 10
Stage III – 11
Stage IV – 12
100 µm

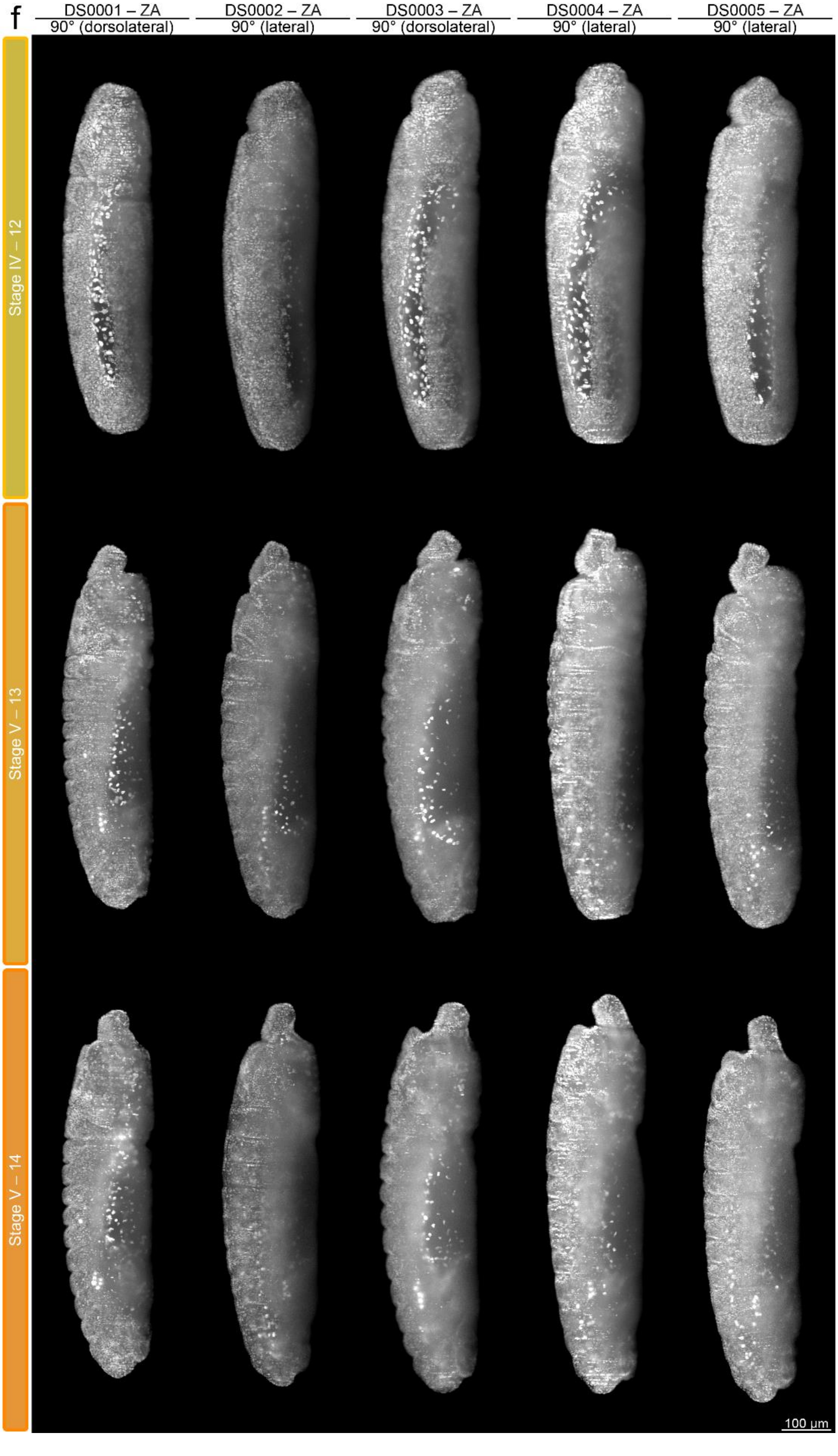

f
DS0001 – ZA
90° (dorsolateral)
DS0002 – ZA
90° (lateral)
DS0003 – ZA
90° (dorsolateral)
DS0004 – ZA
90° (lateral)
DS0005 – ZA
90° (lateral)
Stage IV – 12
Stage V – 13
Stage V – 14
100 µm

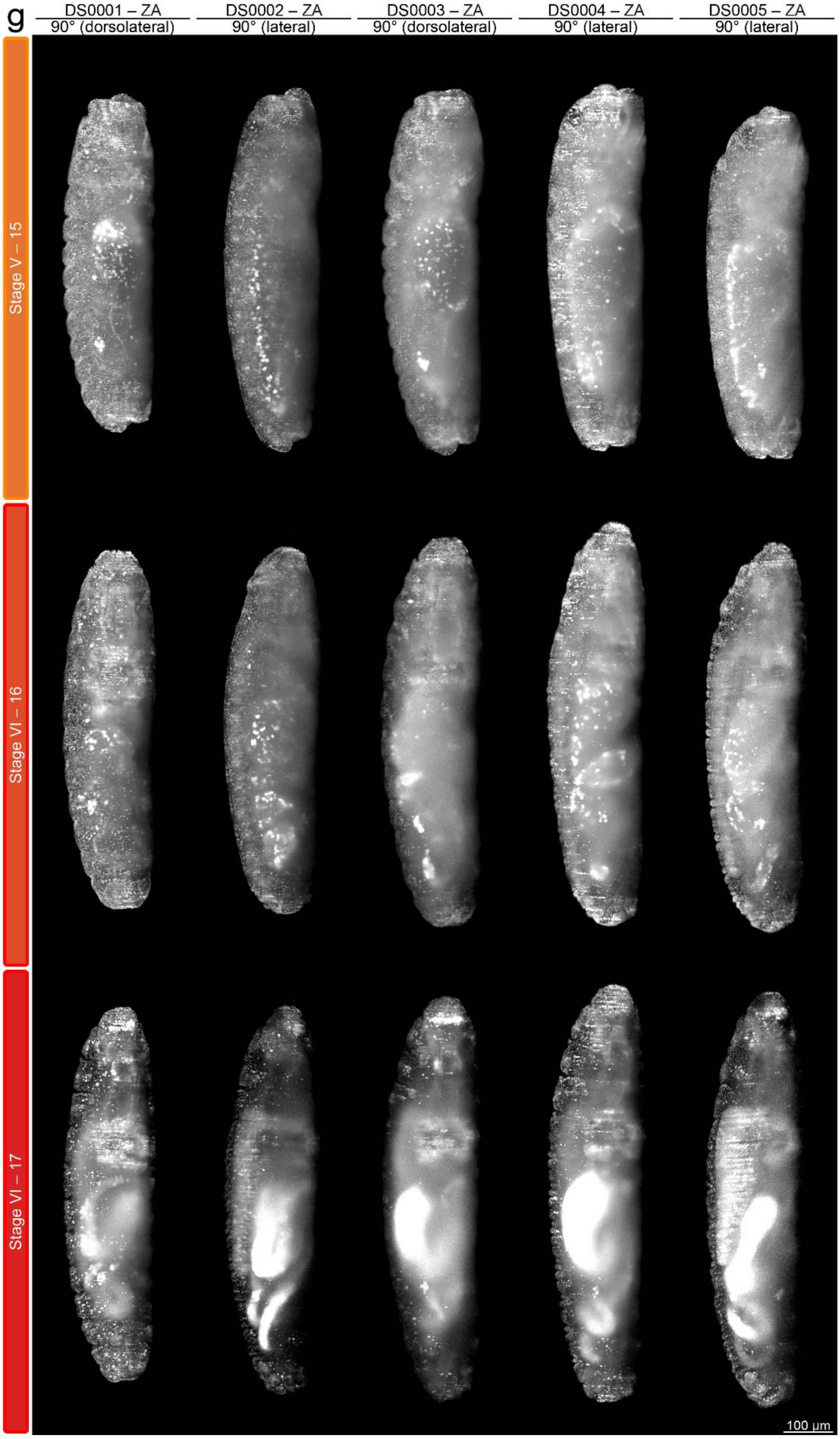

Supplementary Figure 4 – Comparison of datasets DS0001–DS0005 at the onset of the stages (a) I-2 to I-4, (b) I-5 to II-7, (c) III-8, (d) III-8 and III-9, (e) III-10 to IV-12, (f) IV-12 to V-14, (g) V-15 to VI-17. The shown direction changes throughout the figure (at stages III-8 and IV-12), so that characteristic structures for the respective stage can be seen properly. Since the recordings started after the onset of stage I-1, respective image data is not available. Embryos from datasets DS0001 and DS0003 are depicted either ventrolateral or dorsolateral, whereas embryos from DS0002, DS0004 and DS0005 are depicted either ventral, lateral, or dorsal. ZA, *z* maximum projection with image adjustment.

Supplementary Tables

Supplementary Table 1 – Metadata and access information for datasets DS0001–DS0009.

Dataset (DS)	**DS0001**	**DS0002**	**DS0003**
Species	Mediterranean fruit fly (medfly) *Ceratitis capitata* (Wiedemann)<br>Arthropoda → Insecta → Diptera → Tephritidae		
Line	*TREhs43-hid*^{Ala5}_F1m2[52]		
Line Genotype	single insertion / homozygous		
Stock	~50-80 adults, less than 4 weeks old		
Stock Medium	refined sugar (524973, REWE Markt GmbH, Köln, Germany)<br>supplemented with 33% (wt/wt) inactive dry yeast (62-106, Flystuff, San Diego, CA, USA)		
Stock Conditions	12:00 h light / 12:00 h darkness at 25°C and 70% relative humidity<br>(DR-36VL, Percival Scientific, Perry, IA, USA)		
Embryo Collection	cultures were given 10 min at room temperature (23±1°C) to lay embryos		
Pre-Imaging Incubation	embryo mounting procedure, approximately 00:30 h at room temperature (23±1°C),<br>incubation in the sample chamber, approximately 01:30 h at room temperature (23±1°C)<br>resulting in a total of 02:00 h incubation at room temperature (23±1°C)		
LSFM Implementation	sample chamber-based digital scanned laser light sheet fluorescence microscope (DSLM)[3]		
Laser Lines	488 nm / 60 mW Diode Laser<br>(PhoxX 488-60, Omicron-Laserage Laserprodukte GmbH, Rodgau-Dudenhofen, Germany)		
Excitation Objective	2.5× NA 0.06 EC Epiplan-Neofluar objective (422320-9900-000**,** Carl Zeiss, Göttingen, Germany)		
Emission Objective	10× NA 0.3 W N-Achroplan objective (420947-9900-000, Carl Zeiss, Göttingen, Germany)		
Emission Filters	525/50 single-band bandpass filter<br>(FF03-525/50-25, Semrock/AHF Analysentechnik AG, Tübingen, Germany)		
Camera	high-resolution CCD (Clara, Andor, Belfast, United Kingdom), 14 bit, 1040×1392 pixel (pitch 6.45 µm)		
Dataset File Type	TIFF, 16-bit grayscale (planes saved as *z* stacks in ZIP-compressed container files, indicated as PL(ZS))		
Dechorionation	90 s in a 1:9 mixture of<br>~10% (vol/vol) sodium hypochlorite (425044-250ML, Sigma Aldrich, Taufkirchen, Germany)<br>and PBS pH 7.4 (10010-023, Gibco Life Technologies GmbH, Darmstadt, Germany)		
Mounting Method	cobweb holder mounting method (embryos are attached to a thin agarose film spanning a slotted hole)[50]		
Mounting Agarose	1% (wt/vol) low-melt agarose (6351.2, Carl Roth, Karlsruhe, Germany)<br>in PBS pH 7.4 (10010-023, Gibco Life Technologies GmbH, Darmstadt, Germany)		
Imaging Buffer	PBS pH 7.4 (10010-023, Gibco Life Technologies GmbH, Darmstadt, Germany)		
Imaging Temperature	room temperature (23±1°C)	room temperature (23±1°C)	room temperature (23±1°C)
Viewing Perspective	ventrolateral, dorsolateral,<br>dorsolateral, ventrolateral	ventral, lateral,<br>dorsal, lateral	ventrolateral, dorsolateral,<br>dorsolateral, ventrolateral
Developmental Stages	II-2 to VI-17	II-2 to VI-17	II-2 to VI-17
Retrieval	developed to healthy adult	developed to healthy adult	developed to healthy adult

(continued)

Dataset (DS)	**DS0001**	**DS0002**	**DS0003**
Dataset Size	31.1 Gigabyte	31.6 Gigabyte	31.9 Gigabyte
Dataset Access	DOI: 10.5281/zenodo.6448019	DOI: 10.5281/zenodo.6448433	DOI: 10.5281/zenodo.6450798
Figures	2 (first column)	2 (second column)	2 (third column)
Supplementary Figures	3 (first column),<br>4 (first column)	3 (second column),<br>4 (second column)	3 (third column),<br>4 (third column)
Supplementary Videos	1 (first column, first)	1 (first column, second row)	1 (first column third row)
Comment	median development time of datasets DS0001–DS0005	-	-
Time Points (TP)	**121 (TP0001-TP0121)**	**126 (TP0001-TP0126)**	**122 (TP0001-TP0122)**
TP Interval	30 min	30 min	30 min
Total Time ((TP-1)×TP Interval)	60:00 h	62:30 h	60:30 h
Directions (DR)	**4 (DR0001-DR0004)**	**4 (DR0001-DR0004)**	**4 (DR0001-DR0004)**
DR Orientations	0°, 90°, 180°, 270°	0°, 90°, 180°, 270°	0°, 90°, 180°, 270°
Channels (CH)	**1 (CH0001)**	**1 (CH0001)**	**1 (CH0001)**
CH0001 Excitation	488 nm	488 nm	488 nm
CH0001 Power	135 µW (close to the embryo)	135 µW (close to the embryo)	135 µW (close to the embryo)
CH0001 Exposure Time	50 ms	50 ms	50 ms
CH0001 Emission Filter	525/50 single-band bandpass filter	525/50 single-band bandpass filter	525/50 single-band bandpass filter
CH0001 Theoretical Lateral / Axial Resolution	~850 nm / ~3,200 nm	~850 nm / ~3,200 nm	~850 nm / ~3,200 nm
Planes (PL)	**100 (PL0001-PL0100)**	**100 (PL0001-PL0100)**	**100 (PL0001-PL0100)**
Z Spacing	2.58 µm	2.58 µm	2.58 µm
Z Distance (PL×Z Spacing)	258.0 µm	258.0 µm	258.0 µm
X-Dimensions (XD)	**500 pixels**	**500 pixels**	**500 pixels**
X Spacing	0.645 µm	0.645 µm	0.645 µm
X Length (XD×X Spacing)	322.5 µm	322.5 µm	322.5 µm
Y-Dimensions (YD)	**1390 pixels**	**1390 pixels**	**1390 pixels**
Y Spacing	0.645 µm	0.645 µm	0.645 µm
Y Length (YD)×Y Spacing)	896.6 µm	896.6 µm	896.6 µm

(continued)

Dataset (DS)	**DS0004**	**DS0005**	**DS0006**
Species	Mediterranean fruit fly (medfly) *Ceratitis capitata* (Wiedemann)<br>Arthropoda → Insecta → Diptera → Tephritidae		
Line	*TREhs43-hid*^{Ala5}_F1m2[52]		
Line Genotype	single insertion / homozygous		
Stock	~50-80 adults, less than 4 weeks old		
Stock Medium	refined sugar (524973, REWE Markt GmbH, Köln, Germany)<br>supplemented with 33% (wt/wt) inactive dry yeast (62-106, Flystuff, San Diego, CA, USA)		
Stock Conditions	12:00 h light / 12:00 h darkness at 25°C and 70% relative humidity<br>(DR-36VL, Percival Scientific, Perry, IA, USA)		
Embryo Collection	cultures were given 10 min at room temperature (23±1°C) to lay embryos		
Pre-Imaging Incubation	embryo mounting procedure, approximately 00:30 h at room temperature (23±1°C),<br>incubation in the sample chamber, approximately 01:30 h at room temperature (23±1°C)<br>resulting in a total of 02:00 h incubation at room temperature (23±1°C)		
LSFM Implementation	sample chamber-based digital scanned laser light sheet fluorescence microscope (DSLM)[3]		
Laser Lines	488 nm / 60 mW Diode Laser<br>(PhoxX 488-60, Omicron-Laserage Laserprodukte GmbH, Rodgau-Dudenhofen, Germany)		
Excitation Objective	2.5× NA 0.06 EC Epiplan-Neofluar objective (422320-9900-000**,** Carl Zeiss, Göttingen, Germany)		
Emission Objective	10× NA 0.3 W N-Achroplan objective (420947-9900-000, Carl Zeiss, Göttingen, Germany)		
Emission Filters	525/50 single-band bandpass filter<br>(FF03-525/50-25, Semrock/AHF Analysentechnik AG, Tübingen, Germany)		
Camera	high-resolution CCD (Clara, Andor, Belfast, United Kingdom), 14 bit, 1040×1392 pixel (pitch 6.45 µm)		
Dataset File Type	TIFF, 16-bit grayscale (planes saved as *z* stacks in ZIP-compressed container files, indicated as PL(ZS))		
Dechorionation	90 s in a 1:9 mixture of<br>~10% (vol/vol) sodium hypochlorite (425044-250ML, Sigma Aldrich, Taufkirchen, Germany)<br>and PBS pH 7.4 (10010-023, Gibco Life Technologies GmbH, Darmstadt, Germany)		
Mounting Method	cobweb holder mounting method (embryos are attached to a thin agarose film spanning a slotted hole)[50]		
Mounting Agarose	1% (wt/vol) low-melt agarose (6351.2, Carl Roth, Karlsruhe, Germany)<br>in PBS pH 7.4 (10010-023, Gibco Life Technologies GmbH, Darmstadt, Germany)		
Imaging Buffer	PBS pH 7.4 (10010-023, Gibco Life Technologies GmbH, Darmstadt, Germany)		
Imaging Temperature	room temperature (23±1°C)	room temperature (23±1°C)	room temperature (23±1°C)
Viewing Perspective	ventral, lateral,<br>dorsal, lateral	ventral, lateral,<br>dorsal, lateral	ventrolateral, dorsolateral,<br>dorsolateral, ventrolateral
Developmental Stages	II-2 to VI-17	II-2 to VI-17	II-2 to VI-17
Retrieval	developed to healthy adult	developed to healthy adult	did not eclose from the pupa

(continued)

Dataset (DS)	**DS0004**	**DS0005**	**DS0006**
Dataset Size	31.5 Gigabyte	28.9 Gigabyte	28.1 Gigabyte
Dataset Access	DOI: 10.5281/zenodo.6451099	DOI: 10.5281/zenodo.6453980	DOI: 10.5281/zenodo.6455038
Figures	2 (fourth column)	2 (fifth column)	-
Supplementary Figures	3 (fourth column),<br>4 (fourth column)	3 (fifth column),<br>4 (fifth column)	-
Supplementary Videos	1 (second column, first row)	1 (second column, second row)	1 (second column, third row)
Comment	slightly larger than<br>the volume of view along *y*	slightly larger than<br>the volume of view along *y*	excluded<br>from analysis
Time Points (TP)	**116 (TP0001-TP0116)**	**115 (TP0001-TP0115)**	**113 (TP0001-TP0113)**
TP Interval	30 min	30 min	30 min
Total Time<br>*((TP-1)×TP Interval)*	57:30 h	57:00 h	56:00 h
Directions (DR)	**4 (DR0001-DR0004)**	**4 (DR0001-DR0004)**	**4 (DR0001-DR0004)**
DR Orientations	0°, 90°, 180°, 270°	0°, 90°, 180°, 270°	0°, 90°, 180°, 270°
Channels (CH)	**1 (CH0001)**	**1 (CH0001)**	**1 (CH0001)**
CH0001 Excitation	488 nm	488 nm	488 nm
CH0001 Power	135 µW (close to the embryo)	135 µW (close to the embryo)	135 µW (close to the embryo)
CH0001 Exposure Time	50 ms	50 ms	50 ms
CH0001 Emission Filter	525/50 single-band<br>bandpass filter	525/50 single-band<br>bandpass filter	525/50 single-band<br>bandpass filter
CH0001 Theoretical<br>*Lateral / Axial Resolution*	~850 nm / ~3,200 nm	~850 nm / ~3,200 nm	~850 nm / ~3,200 nm
Planes (PL)	**100 (PL0001-PL0100)**	**100 (PL0001-PL0100)**	**100 (PL0001-PL0100)**
Z Spacing	2.58 µm	2.58 µm	2.58 µm
Z Distance<br>*(PL×Z Spacing)*	258.0 µm	258.0 µm	258.0 µm
X-Dimensions (XD)	**500 pixels**	**500 pixels**	**500 pixels**
X Spacing	0.645 µm	0.645 µm	0.645 µm
X Length<br>*(XD×X Spacing)*	322.5 µm	322.5 µm	322.5 µm
Y-Dimensions (YD)	**1390 pixels**	**1390 pixels**	**1390 pixels**
Y Spacing	0.645 µm	0.645 µm	0.645 µm
Y Length<br>*(YD)×Y Spacing)*	896.6 µm	896.6 µm	896.6 µm

(continued)

Dataset (DS)	**DS0007**	**DS0008**	**DS0009**
Species	Mediterranean fruit fly (medfly) *Ceratitis capitata* (Wiedemann)<br>Arthropoda → Insecta → Diptera → Tephritidae		
Line	*TREhs43-hid*^{Ala5}_F1m2[52]		
Line Genotype	single insertion / homozygous		
Stock	~50-80 adults, less than 4 weeks old		
Stock Medium	refined sugar (524973, REWE Markt GmbH, Köln, Germany)<br>supplemented with 33% (wt/wt) inactive dry yeast (62-106, Flystuff, San Diego, CA, USA)		
Stock Conditions	12:00 h light / 12:00 h darkness at 25°C and 70% relative humidity<br>(DR-36VL, Percival Scientific, Perry, IA, USA)		
Embryo Collection	cultures were given 10 min at room temperature (23±1°C) to lay embryos		
Pre-Imaging Incubation	embryo mounting procedure, approximately 00:30 h at room temperature (23±1°C),<br>incubation in the sample chamber, approximately 01:30 h at room temperature (23±1°C)<br>resulting in a total of 02:00 h incubation at room temperature (23±1°C)		
LSFM Implementation	sample chamber-based digital scanned laser light sheet fluorescence microscope (DSLM)[3]		
Laser Lines	488 nm / 60 mW Diode Laser<br>(PhoxX 488-60, Omicron-Laserage Laserprodukte GmbH, Rodgau-Dudenhofen, Germany)		
Excitation Objective	2.5× NA 0.06 EC Epiplan-Neofluar objective (422320-9900-000**,** Carl Zeiss, Göttingen, Germany)		
Emission Objective	20× NA 0.5 W N-Achroplan objective (420957-9900-000, Carl Zeiss, Göttingen, Germany)		
Emission Filters	525/50 single-band bandpass filter<br>(FF03-525/50-25, Semrock/AHF Analysentechnik AG, Tübingen, Germany)		
Camera	high-resolution CCD (Clara, Andor, Belfast, United Kingdom), 14 bit, 1040×1392 pixel (pitch 6.45 µm)		
Dataset File Type	TIFF, 16-bit grayscale (planes saved as *z* stacks in ZIP-compressed container files, indicated as PL(ZS))		
Dechorionation	90 s in a 1:9 mixture of<br>~10% (vol/vol) sodium hypochlorite (425044-250ML, Sigma Aldrich, Taufkirchen, Germany)<br>and PBS pH 7.4 (10010-023, Gibco Life Technologies GmbH, Darmstadt, Germany)		
Mounting Method	cobweb holder mounting method (embryos are attached to a thin agarose film spanning a slotted hole)[50]		
Mounting Agarose	1% (wt/vol) low-melt agarose (6351.2, Carl Roth, Karlsruhe, Germany)<br>in PBS pH 7.4 (10010-023, Gibco Life Technologies GmbH, Darmstadt, Germany)		
Imaging Buffer	PBS pH 7.4 (10010-023, Gibco Life Technologies GmbH, Darmstadt, Germany)		
Imaging Temperature	room temperature (23±1°C)	room temperature (23±1°C)	room temperature (23±1°C)
Viewing Perspective	ventral, lateral,<br>dorsal, lateral	ventral, lateral,<br>dorsal, lateral	anterior, lateral
Developmental Stages	II-2 to IV-12	II-2 to VI-17	II-2 to VI-16
Retrieval	developed to healthy adult	developed to healthy adult	developed to healthy adult

(continued)

Dataset (DS)	**DS0007**	**DS0008**	**DS0009**
Dataset Size	19.4 Gigabyte (TIFF)	41.7 Gigabyte (TIFF)	11.8 Gigabyte (TIFF)
Dataset Access	DOI: 10.5281/zenodo.6456820	DOI: 10.5281/zenodo.6457004	DOI: 10.5281/zenodo.6457894
Figures	-	-	-
Supplementary Figures	-	-	-
Supplementary Videos	-	-	-
Comment	embryo not captured *in toto*, covers germ cell formation	embryo not captured *in toto*, covers head involution	embryo not captured *in toto*, agarose hemisphere mounting
Time Points (TP)	**49 (TP0001-TP0049)**	**123 (TP0001-TP0123)**	**93 (TP0001-TP0093)**
TP Interval	30 min	30 min	30 min
Total Time ((TP-1)×TP Interval)	24:00 h	61:00 h	46:00 h
Directions (DR)	**4 (DR0001-DR0004)**	**4 (DR0001-DR0004)**	**2 (DR0001-DR0002)**
DR Orientations	0°, 90°, 180°, 270°	0°, 90°, 180°, 270°	0°, 90°
Channels (CH)	**1 (CH0001)**	**1 (CH0001)**	**1 (CH0001)**
CH0001 Excitation	488 nm	488 nm	488 nm
CH0001 Power	135 µW (close to the embryo)	135 µW (close to the embryo)	135 µW (close to the embryo)
CH0001 Exposure Time	50 ms	50 ms	50 ms
CH0001 Emission Filter	525/50 single-band bandpass filter	525/50 single-band bandpass filter	525/50 single-band bandpass filter
CH0001 Theoretical Lateral / Axial Resolution	~510 nm / ~2,300 nm	~510 nm / ~2,300 nm	~510 nm / ~2,300 nm
Planes (PL)	**100 (PL0001-PL0100)**	**100 (PL0001-PL0100)**	**DR0001: 115 (PL0001-PL0115), DR0002: 100 (PL0001-PL0100)**
Z Spacing	2.58 µm	2.58 µm	2.58 µm
Z Distance (PL×Z Spacing)	258.0 µm	258.0 µm	DR0001: 296.7 µm, DR0002: 258.0 µm
X-Dimensions (XD)	**720 pixels**	**720 pixels**	**DR0001: 720 pixels, DR0002: 1000 pixels**
X Spacing	0.3225 µm	0.3225 µm	0.3225 µm
X Length (XD×X Spacing)	232.2 µm	232.2 µm	DR0001: 232.2 µm, DR0002: 322.5 µm
Y-Dimensions (YD)	**1300 pixels**	**1300 pixels**	**720 pixels**
Y Spacing	0.3225 µm	0.3225 µm	0.3225 µm
Y Length (YD)×Y Spacing)	419.25 µm	419.25 µm	232.2 µm

Supplementary Table 2 – Embryogenetic events onset time points for DS0001–DS0005. Embryogenetic events are color-coded (see Methods section). Since imaging started approximately 2 h after embryo collection, the first time point (TP0001) was set to 02:00 h of absolute development. I, blastoderm formation; II, early gastrulation; III, germband elongation; IV, germband retraction; V, dorsal closure; VI, muscular movement. TP, imaging time point; time, absolute time passed from the onset of I until the indicated TP; rel dev, relative progress of embryonic development from the onset of I until the indicated TP.

Event	DS0001 (median)			DS0002 (longer)			DS0003 (longer)		
	TP	time	rel dev	TP	time	rel dev	TP	time	rel dev
I	-	00:00 h	0.0%	-	00:00 h	0.0%	-	00:00 h	0.0%
II	0020	11:30 h	18.4%	0022	12:30 h	19.2%	0021	12:00 h	19.0%
III	0026	14:30 h	23.2%	0027	15:00 h	23.1%	0026	14:30 h	23.0%
IV	0046	24:30 h	39.2%	0048	25:30 h	39.2%	0046	24:30 h	38.9%
V	0061	32:00 h	51.2%	0062	32:30 h	50.0%	0059	31:00 h	49.2%
VI	0087	45:00 h	72.0%	0084	43:30 h	66.9%	0086	44:30 h	70.6%
Hatch	0122	62:30 h	100.0%	0127	65:00 h	100.0%	0123	63:00 h	100.0%

(continued)

Event	DS0004 (shorter)			DS0005 (shorter)			Standard Deviations	
	TP	time	rel dev	TP	time	rel dev	time	rel dev
I	-	00:00 h	0.0%	-	00:00 h	0.0%	±00:00 h	±0.0%
II	0019	11:00 h	18.3%	0019	11:00 h	18.5%	±00:39 h	±0.4%
III	0024	13:30 h	22.5%	0024	13:30 h	22.7%	±00:40 h	±0.3%
IV	0044	23:30 h	39.2%	0044	23:30 h	39.5%	±00:50 h	±0.2%
V	0053	28:00 h	46.7%	0055	29:00 h	48.7%	±01:56 h	±1.7%
VI	0079	41:00 h	68.3%	0082	42:30 h	71.4%	±01:36 h	±2.2%
Hatch	0117	60:00 h	100.0%	0116	59:30 h	100.0%	±02:15 h	±0.0%

Supplementary Table 3 – Stage onset time points for DS0001–DS0005. Onset time points of embryogenetic events are color-coded (see Methods section). Since imaging started approximately 2 h after embryo collection, the first time point (TP0001) was set to 02:00 h of absolute development. TP, imaging time point; time, absolute time passed from the onset of I-1 until the indicated TP; rel dev, relative progress of embryonic development from the onset of I-1 until the indicated TP.

Stage	DS0001 (median)			DS0002 (longer)			DS0003 (longer)		
	TP	time	rel dev	TP	time	rel dev	TP	time	rel dev
**I-1**	-	00:00 h	0.0%	-	00:00 h	0.0%	-	00:00 h	0.0%
**I-2**	0001	02:00 h	3.2%	0001	02:00 h	3.1%	0001	02:00 h	3.2%
**I-3**	0004	03:30 h	5.6%	0004	03:30 h	5.4%	0004	03:30 h	5.6%
**I-4**	0006	04:30 h	7.2%	0007	05:00 h	7.7%	0006	04:30 h	7.1%
**I-5**	0010	06:30 h	10.4%	0011	07:00 h	10.8%	0010	06:30 h	10.3%
**II-6**	0020	11:30 h	18.4%	0022	12:30 h	19.2%	0021	12:00 h	19.0%
**II-7**	0024	13:30 h	21.6%	0026	14:30 h	22.3%	0025	14:00 h	22.2%
**III-8**	0026	14:30 h	23.2%	0027	15:00 h	23.1%	0026	14:30 h	23.0%
**III-9**	0030	16:30 h	26.4%	0031	17:00 h	26.2%	0030	16:30 h	26.2%
**III-10**	0033	18:00 h	28.8%	0032	17:30 h	26.9%	0032	17:30 h	27.8%
**III-11**	0041	22:00 h	35.2%	0043	23:00 h	35.4%	0038	20:30 h	32.5%
**IV-12**	0046	24:30 h	39.2%	0048	25:30 h	39.2%	0046	24:30 h	38.9%
**V-13**	0061	32:00 h	51.2%	0062	32:30 h	50.0%	0059	31:00 h	49.2%
**V-14**	0066	34:30 h	55.2%	0068	35:30 h	54.6%	0066	34:30 h	54.8%
**V-15**	0073	38:00 h	60.8%	0074	38:30 h	59.2%	0071	37:00 h	58.7%
**VI-16**	0087	45:00 h	72.0%	0084	43:30 h	66.9%	0086	44:30 h	70.6%
**VI-17**	0106	54:30 h	87.2%	0110	56:30 h	86.9%	0104	53:30 h	84.9%
**Hatch**	0122	62:30 h	100.0%	0127	65:00 h	100.0%	0123	63:00 h	100.0%

(continued)

Stage	DS0004 (shorter)			DS0005 (shorter)			Standard Deviations	
	TP	time	rel dev	TP	time	rel dev	time	rel dev
**I-1**	-	00:00 h	0.0%	-	00:00 h	0.0%	±00:00 h	±0.0%
**I-2**	0001	02:00 h	3.3%	0001	02:00 h	3.4%	±00:00 h	±0.1%
**I-3**	0004	03:30 h	5.8%	0004	03:30 h	5.9%	±00:00 h	±0.2%
**I-4**	0006	04:30 h	7.5%	0006	04:30 h	7.6%	±00:13 h	±0.2%
**I-5**	0010	06:30 h	10.8%	0010	06:30 h	10.9%	±00:13 h	±0.3%
**II-6**	0019	11:00 h	18.3%	0019	11:00 h	18.5%	±00:39 h	±0.4%
**II-7**	0023	13:00 h	21.7%	0023	13:00 h	21.8%	±00:39 h	±0.3%
**III-8**	0024	13:30 h	22.5%	0024	13:30 h	22.7%	±00:40 h	±0.3%
**III-9**	0029	16:00 h	26.7%	0029	16:00 h	26.9%	±00:25 h	±0.3%
**III-10**	0031	17:00 h	28.3%	0031	17:00 h	28.6%	±00:25 h	±0.8%
**III-11**	0036	19:30 h	32.5%	0038	20:30 h	34.5%	±01:23 h	±1.4%
**IV-12**	0044	23:30 h	39.2%	0044	23:30 h	39.5%	±00:50 h	±0.2%
**V-13**	0053	28:00 h	46.7%	0055	29:00 h	48.7%	±01:56 h	±1.7%
**V-14**	0062	32:30 h	54.2%	0062	32:30 h	54.6%	±01:20 h	±0.4%
**V-15**	0069	36:00 h	60.0%	0071	37:00 h	62.2%	±00:58 h	±1.4%
**VI-16**	0079	41:00 h	68.3%	0082	42:30 h	71.4%	±01:36 h	±2.2%
**VI-17**	0097	50:00 h	83.3%	0101	52:00 h	87.4%	±02:27 h	±1.8%
**Hatch**	0117	60:00 h	100.0%	0116	59:30 h	100.0%	±02:15 h	±0.0%

Supplementary Table 4 – Time point-based alignment of DS0002–DS0005 to DS0001. Onset time points of embryogenetic events are color-coded (see Methods section). Since imaging started approximately 2 h after embryo collection, the first time point (TP0001) was set to 02:00 h of absolute development. Imaging typically begins with stage I-2, the values for I-1 are extrapolated. TP, imaging time point; time, absolute time passed from the onset of I-1 until the indicated TP; rel dev, relative progress of embryonic development from the onset of I-1 until the indicated TP.

Stage	TP	DS0001 (median)		DS0002 (aligned)		DS0003 (aligned)		DS0004 (aligned)		DS0005 (aligned)		Standard Deviation	
		time	rel dev	time	rel dev	time	rel dev	time	rel dev	time	rel dev	time	rel dev
**I-1**	-	00:00 h	0.0%	00:00 h	0.0%	00:00 h	0.0%	00:00 h	0.0%	00:00 h	0.0%	±00:00 h	±0.0%
	-	00:30 h	0.8%	00:30 h	0.8%	00:30 h	0.8%	00:30 h	0.8%	00:30 h	0.8%	±00:00 h	±0.0%
	-	01:00 h	1.6%	01:00 h	1.5%	01:00 h	1.6%	01:00 h	1.7%	01:00 h	1.7%	±00:00 h	±0.1%
	-	01:30 h	2.4%	01:30 h	2.3%	01:30 h	2.4%	01:30 h	2.5%	01:30 h	2.5%	±00:00 h	±0.1%
**I-2**	**0001**	02:00 h	3.2%	02:00 h	3.1%	02:00 h	3.2%	02:00 h	3.3%	02:00 h	3.4%	±00:00 h	±0.1%
	**0002**	02:30 h	4.0%	02:30 h	3.8%	02:30 h	4.0%	02:30 h	4.2%	02:30 h	4.2%	±00:00 h	±0.1%
	**0003**	03:00 h	4.8%	03:00 h	4.6%	03:00 h	4.8%	03:00 h	5.0%	03:00 h	5.0%	±00:00 h	±0.2%
**I-3**	**0004**	03:30 h	5.6%	03:30 h	5.4%	03:30 h	5.6%	03:30 h	5.8%	03:30 h	5.9%	±00:00 h	±0.2%
	**0005**	04:00 h	6.4%	04:30 h	6.9%	04:00 h	6.3%	04:00 h	6.7%	04:00 h	6.7%	±00:13 h	±0.2%
**I-4**	**0007**	04:30 h	7.2%	05:00 h	7.7%	04:30 h	7.1%	04:30 h	7.5%	04:30 h	7.6%	±00:13 h	±0.2%
	**0008**	05:00 h	8.0%	05:30 h	8.5%	05:00 h	7.9%	05:00 h	8.3%	05:00 h	8.4%	±00:13 h	±0.2%
	**0009**	05:30 h	8.8%	06:00 h	9.2%	05:30 h	8.7%	05:30 h	9.2%	05:30 h	9.2%	±00:13 h	±0.2%
	**0010**	06:00 h	9.6%	06:30 h	10.0%	06:00 h	9.5%	06:00 h	10.0%	06:00 h	10.1%	±00:13 h	±0.3%
**I-5**	**0011**	06:30 h	10.4%	07:00 h	10.8%	06:30 h	10.3%	06:30 h	10.8%	06:30 h	10.9%	±00:13 h	±0.3%
	**0012**	07:00 h	11.2%	07:33 h	11.6%	07:03 h	11.2%	06:56 h	11.6%	06:56 h	11.7%	±00:15 h	±0.2%
	**0013**	07:30 h	12.0%	08:06 h	12.5%	07:36 h	12.1%	07:23 h	12.3%	07:23 h	12.4%	±00:18 h	±0.2%
	**0014**	08:00 h	12.8%	08:40 h	13.3%	08:10 h	13.0%	07:50 h	13.1%	07:50 h	13.2%	±00:20 h	±0.2%
	**0015**	08:30 h	13.6%	09:13 h	14.2%	08:43 h	13.8%	08:16 h	13.8%	08:16 h	13.9%	±00:23 h	±0.2%
	**0016**	09:00 h	14.4%	09:46 h	15.0%	09:16 h	14.7%	08:43 h	14.5%	08:43 h	14.7%	±00:26 h	±0.2%
	**0017**	09:30 h	15.2%	10:20 h	15.9%	09:50 h	15.6%	09:10 h	15.3%	09:10 h	15.4%	±00:29 h	±0.3%
	**0018**	10:00 h	16.0%	10:53 h	16.8%	10:23 h	16.5%	09:36 h	16.0%	09:36 h	16.2%	±00:32 h	±0.3%
	**0019**	10:30 h	16.8%	11:26 h	17.6%	10:56 h	17.4%	10:03 h	16.8%	10:03 h	16.9%	±00:35 h	±0.4%
	**0020**	11:00 h	17.6%	12:00 h	18.5%	11:30 h	18.3%	10:30 h	17.5%	10:30 h	17.6%	±00:39 h	±0.4%
**II-6**	**0021**	11:30 h	18.4%	12:30 h	19.2%	12:00 h	19.0%	11:00 h	18.3%	11:00 h	18.5%	±00:39 h	±0.4%
	**0022**	12:00 h	19.2%	13:00 h	20.0%	12:30 h	19.8%	11:30 h	19.2%	11:30 h	19.3%	±00:39 h	±0.4%
	**0023**	12:30 h	20.0%	13:30 h	20.8%	13:00 h	20.6%	12:00 h	20.0%	12:00 h	20.2%	±00:39 h	±0.4%
	**0024**	13:00 h	20.8%	14:00 h	21.5%	13:30 h	21.4%	12:30 h	20.8%	12:30 h	21.0%	±00:39 h	±0.3%
**II-7**	**0024**	13:30 h	21.6%	14:30 h	22.3%	14:00 h	22.2%	13:00 h	21.7%	13:00 h	21.8%	±00:39 h	±0.3%
	**0025**	14:00 h	22.4%	14:30 h	22.3%	14:00 h	22.2%	13:00 h	21.7%	13:00 h	21.8%	±00:40 h	±0.3%
**III-8**	**0026**	14:30 h	23.2%	15:00 h	23.1%	14:30 h	23.0%	13:30 h	22.5%	13:30 h	22.7%	±00:40 h	±0.3%
	**0027**	15:00 h	24.0%	15:30 h	23.8%	15:00 h	23.8%	14:10 h	23.6%	14:10 h	23.8%	±00:35 h	±0.1%
	**0028**	15:30 h	24.8%	16:00 h	24.6%	15:30 h	24.6%	14:50 h	24.7%	14:50 h	24.9%	±00:30 h	±0.1%
	**0029**	16:00 h	25.6%	16:30 h	25.4%	16:00 h	25.4%	15:30 h	25.8%	15:30 h	26.1%	±00:25 h	±0.3%
**III-9**	**0030**	16:30 h	26.4%	17:00 h	26.2%	16:30 h	26.2%	16:00 h	26.7%	16:00 h	26.9%	±00:25 h	±0.3%
	**0031**	17:00 h	27.2%	17:00 h	26.2%	16:45 h	26.6%	16:15 h	27.1%	16:15 h	27.3%	±00:22 h	±0.5%
	**0032**	17:30 h	28.0%	17:00 h	26.2%	17:00 h	27.0%	16:30 h	27.5%	16:30 h	27.7%	±00:25 h	±0.7%
**III-10**	**0033**	18:00 h	28.8%	17:30 h	26.9%	17:30 h	27.8%	17:00 h	28.3%	17:00 h	28.6%	±00:25 h	±0.8%
	**0034**	18:30 h	29.6%	18:12 h	28.0%	17:51 h	28.3%	17:17 h	28.8%	17:25 h	29.3%	±00:30 h	±0.7%
	**0035**	19:00 h	30.4%	18:55 h	29.1%	18:12 h	28.9%	17:34 h	29.3%	17:51 h	30.0%	±00:38 h	±0.6%
	**0036**	19:30 h	31.2%	19:38 h	30.2%	18:34 h	29.5%	17:51 h	29.8%	18:17 h	30.7%	±00:46 h	±0.7%
	**0037**	20:00 h	32.0%	20:21 h	31.3%	18:55 h	30.0%	18:08 h	30.2%	18:42 h	31.5%	±00:55 h	±0.8%
	**0038**	20:30 h	32.8%	21:04 h	32.4%	19:17 h	30.6%	18:25 h	30.7%	19:08 h	32.2%	±01:04 h	±1.0%
	**0039**	21:00 h	33.6%	21:47 h	33.5%	19:38 h	31.2%	18:42 h	31.2%	19:34 h	32.9%	±01:13 h	±1.2%
	**0040**	21:30 h	34.4%	22:30 h	34.6%	20:00 h	31.7%	19:00 h	31.7%	20:00 h	33.6%	±01:23 h	±1.4%
**III-11**	**0041**	22:00 h	35.2%	23:00 h	35.4%	20:30 h	32.5%	19:30 h	32.5%	20:30 h	34.5%	±01:23 h	±1.4%
	**0042**	22:30 h	36.0%	23:30 h	36.2%	21:22 h	33.9%	20:22 h	34.0%	21:07 h	35.5%	±01:13 h	±1.1%
	**0043**	23:00 h	36.8%	24:00 h	36.9%	22:15 h	35.3%	21:15 h	35.4%	21:45 h	36.6%	±01:04 h	±0.8%
	**0044**	23:30 h	37.6%	24:30 h	37.7%	23:07 h	36.7%	22:07 h	36.9%	22:22 h	37.6%	±00:56 h	±0.5%
	**0045**	24:00 h	38.4%	25:00 h	38.5%	24:00 h	38.1%	23:00 h	38.3%	23:00 h	38.7%	±00:50 h	±0.2%
**IV-12**	**0046**	24:30 h	39.2%	25:30 h	39.2%	24:30 h	38.9%	23:30 h	39.2%	23:30 h	39.5%	±00:50 h	±0.2%
	**0047**	25:00 h	40.0%	25:57 h	39.9%	24:55 h	39.6%	23:47 h	39.6%	23:51 h	40.1%	±00:54 h	±0.2%
	**0048**	25:30 h	40.8%	26:25 h	40.7%	25:21 h	40.2%	24:04 h	40.1%	24:12 h	40.7%	±00:58 h	±0.3%
	**0049**	26:00 h	41.6%	26:53 h	41.4%	25:47 h	40.9%	24:21 h	40.6%	24:34 h	41.3%	±01:03 h	±0.4%
	**0050**	26:30 h	42.4%	27:21 h	42.1%	26:12 h	41.6%	24:38 h	41.1%	24:55 h	41.9%	±01:07 h	±0.5%
	**0051**	27:00 h	43.2%	27:49 h	42.8%	26:38 h	42.3%	24:55 h	41.5%	25:17 h	42.5%	±01:12 h	±0.6%
	**0052**	27:30 h	44.0%	28:17 h	43.5%	27:04 h	43.0%	25:12 h	42.0%	25:38 h	43.1%	±01:17 h	±0.7%
	**0053**	28:00 h	44.8%	28:45 h	44.2%	27:30 h	43.7%	25:30 h	42.5%	26:00 h	43.7%	±01:21 h	±0.9%
	**0054**	28:30 h	45.6%	29:12 h	44.9%	27:55 h	44.3%	25:47 h	43.0%	26:21 h	44.3%	±01:26 h	±1.0%
	**0055**	29:00 h	46.4%	29:40 h	45.7%	28:21 h	45.0%	26:04 h	43.5%	26:42 h	44.9%	±01:31 h	±1.1%
	**0056**	29:30 h	47.2%	30:08 h	46.4%	28:47 h	45.7%	26:21 h	43.9%	27:04 h	45.5%	±01:36 h	±1.2%
	**0057**	30:00 h	48.0%	30:36 h	47.1%	29:12 h	46.4%	26:38 h	44.4%	27:25 h	46.1%	±01:41 h	±1.3%
	**0058**	30:30 h	48.8%	31:04 h	47.8%	29:38 h	47.1%	26:55 h	44.9%	27:47 h	46.7%	±01:46 h	±1.5%
	**0059**	31:00 h	49.6%	31:32 h	48.5%	30:04 h	47.7%	27:12 h	45.4%	28:08 h	47.3%	±01:51 h	±1.6%
	**0060**	31:30 h	50.4%	32:00 h	49.2%	30:30 h	48.4%	27:30 h	45.8%	28:30 h	47.9%	±01:56 h	±1.7%
**V-13**	**0061**	32:00 h	51.2%	32:30 h	50.0%	31:00 h	49.2%	28:00 h	46.7%	29:00 h	48.7%	±01:56 h	±1.7%
	**0062**	32:30 h	52.0%	33:07 h	51.0%	31:45 h	50.4%	29:00 h	48.3%	29:45 h	50.0%	±01:46 h	±1.3%
	**0063**	33:00 h	52.8%	33:45 h	51.9%	32:30 h	51.6%	30:00 h	50.0%	30:30 h	51.3%	±01:37 h	±1.0%
	**0064**	33:30 h	53.6%	34:22 h	52.9%	33:15 h	52.8%	31:00 h	51.7%	31:15 h	52.5%	±01:28 h	±0.7%
	**0065**	34:00 h	54.4%	35:00 h	53.8%	34:00 h	54.0%	32:00 h	53.3%	32:00 h	53.8%	±01:20 h	±0.4%
**V-14**	**0066**	34:30 h	55.2%	35:30 h	54.6%	34:30 h	54.8%	32:30 h	54.2%	32:30 h	54.6%	±01:20 h	±0.4%
	**0067**	35:00 h	56.0%	35:55 h	55.3%	34:50 h	55.3%	33:00 h	55.0%	33:10 h	55.7%	±01:15 h	±0.4%
	**0068**	35:30 h	56.8%	36:20 h	55.9%	35:10 h	55.8%	33:30 h	55.8%	33:50 h	56.9%	±01:10 h	±0.5%
	**0069**	36:00 h	57.6%	36:45 h	56.5%	35:30 h	56.3%	34:00 h	56.7%	34:30 h	58.0%	±01:06 h	±0.7%
	**0070**	36:30 h	58.4%	37:10 h	57.2%	35:50 h	56.9%	34:30 h	57.5%	35:10 h	59.1%	±01:03 h	±0.9%
	**0071**	37:00 h	59.2%	37:35 h	57.8%	36:10 h	57.4%	35:00 h	58.3%	35:50 h	60.2%	±01:00 h	±1.1%
	**0072**	37:30 h	60.0%	38:00 h	58.5%	36:30 h	57.9%	35:30 h	59.2%	36:30 h	61.3%	±00:58 h	±1.3%

(continued)

**V-15**	**0073**	38:00 h	60.8%	38:30 h	59.2%	37:00 h	58.7%	36:00 h	60.0%	37:00 h	62.2%	±00:58 h	±1.4%
	**0074**	38:30 h	61.6%	38:50 h	59.8%	37:32 h	59.6%	36:20 h	60.6%	37:23 h	62.8%	±00:59 h	±1.4%
	**0075**	39:00 h	62.4%	39:11 h	60.3%	38:04 h	60.4%	36:41 h	61.2%	37:46 h	63.5%	±01:00 h	±1.4%
	**0076**	39:30 h	63.2%	39:32 h	60.8%	38:36 h	61.3%	37:02 h	61.7%	38:09 h	64.1%	±01:02 h	±1.4%
	**0077**	40:00 h	64.0%	39:53 h	61.4%	39:09 h	62.1%	37:23 h	62.3%	38:32 h	64.8%	±01:04 h	±1.4%
	**0078**	40:30 h	64.8%	40:13 h	61.9%	39:41 h	63.0%	37:43 h	62.9%	38:55 h	65.4%	±01:07 h	±1.5%
	**0079**	41:00 h	65.6%	40:34 h	62.4%	40:13 h	63.9%	38:04 h	63.5%	39:18 h	66.1%	±01:09 h	±1.5%
	**0080**	41:30 h	66.4%	40:55 h	63.0%	40:46 h	64.7%	38:25 h	64.0%	39:41 h	66.7%	±01:13 h	±1.6%
	**0081**	42:00 h	67.2%	41:16 h	63.5%	41:18 h	65.6%	38:46 h	64.6%	40:04 h	67.4%	±01:16 h	±1.7%
	**0082**	42:30 h	68.0%	41:36 h	64.0%	41:50 h	66.4%	39:06 h	65.2%	40:27 h	68.0%	±01:20 h	±1.7%
	**0083**	43:00 h	68.8%	41:57 h	64.6%	42:23 h	67.3%	39:27 h	65.8%	40:50 h	68.6%	±01:23 h	±1.8%
	**0084**	43:30 h	69.6%	42:18 h	65.1%	42:55 h	68.1%	39:48 h	66.3%	41:13 h	69.3%	±01:27 h	±1.9%
	**0085**	44:00 h	70.4%	42:39 h	65.6%	43:27 h	69.0%	40:09 h	66.9%	41:36 h	69.9%	±01:32 h	±2.0%
	**0086**	44:30 h	71.2%	43:00 h	66.2%	44:00 h	69.8%	40:30 h	67.5%	42:00 h	70.6%	±01:36 h	±2.1%
**VI-16**	**0087**	45:00 h	72.0%	43:30 h	66.9%	44:30 h	70.6%	41:00 h	68.3%	42:30 h	71.4%	±01:36 h	±2.2%
	**0088**	45:30 h	72.8%	44:11 h	68.0%	44:58 h	71.4%	41:28 h	69.1%	43:00 h	72.3%	±01:37 h	±2.1%
	**0089**	46:00 h	73.6%	44:53 h	69.1%	45:26 h	72.1%	41:56 h	69.9%	43:30 h	73.1%	±01:38 h	±2.0%
	**0090**	46:30 h	74.4%	45:35 h	70.1%	45:55 h	72.9%	42:25 h	70.7%	44:00 h	73.9%	±01:39 h	±1.9%
	**0091**	47:00 h	75.2%	46:16 h	71.2%	46:23 h	73.6%	42:53 h	71.5%	44:30 h	74.8%	±01:41 h	±1.8%
	**0092**	47:30 h	76.0%	46:58 h	72.3%	46:51 h	74.4%	43:21 h	72.3%	45:00 h	75.6%	±01:43 h	±1.8%
	**0093**	48:00 h	76.8%	47:40 h	73.3%	47:20 h	75.1%	43:50 h	73.1%	45:30 h	76.5%	±01:45 h	±1.7%
	**0094**	48:30 h	77.6%	48:21 h	74.4%	47:48 h	75.9%	44:18 h	73.8%	46:00 h	77.3%	±01:48 h	±1.7%
	**0095**	49:00 h	78.4%	49:03 h	75.5%	48:16 h	76.6%	44:46 h	74.6%	46:30 h	78.2%	±01:50 h	±1.6%
	**0096**	49:30 h	79.2%	49:45 h	76.5%	48:45 h	77.4%	45:15 h	75.4%	47:00 h	79.0%	±01:53 h	±1.6%
	**0097**	50:00 h	80.0%	50:26 h	77.6%	49:13 h	78.1%	45:43 h	76.2%	47:30 h	79.8%	±01:57 h	±1.6%
	**0098**	50:30 h	80.8%	51:08 h	78.7%	49:41 h	78.9%	46:11 h	77.0%	48:00 h	80.7%	±02:00 h	±1.6%
	**0099**	51:00 h	81.6%	51:50 h	79.7%	50:10 h	79.6%	46:40 h	77.8%	48:30 h	81.5%	±02:03 h	±1.6%
	**0100**	51:30 h	82.4%	52:31 h	80.8%	50:38 h	80.4%	47:08 h	78.6%	49:00 h	82.4%	±02:07 h	±1.6%
	**0101**	52:00 h	83.2%	53:13 h	81.9%	51:06 h	81.1%	47:36 h	79.4%	49:30 h	83.2%	±02:11 h	±1.6%
	**0102**	52:30 h	84.0%	53:55 h	82.9%	51:35 h	81.9%	48:05 h	80.1%	50:00 h	84.0%	±02:15 h	±1.6%
	**0103**	53:00 h	84.8%	54:36 h	84.0%	52:03 h	82.6%	48:33 h	80.9%	50:30 h	84.9%	±02:19 h	±1.7%
	**0104**	53:30 h	85.6%	55:18 h	85.1%	52:31 h	83.4%	49:01 h	81.7%	51:00 h	85.7%	±02:23 h	±1.7%
	**0105**	54:00 h	86.4%	56:00 h	86.2%	53:00 h	84.1%	49:30 h	82.5%	51:30 h	86.6%	±02:27 h	±1.8%
**VI-17**	**0106**	54:30 h	87.2%	56:30 h	86.9%	53:30 h	84.9%	50:00 h	83.3%	52:00 h	87.4%	±02:27 h	±1.8%
	**0107**	55:00 h	88.0%	57:02 h	87.7%	54:06 h	85.9%	50:38 h	84.4%	52:28 h	88.2%	±02:26 h	±1.7%
	**0108**	55:30 h	88.8%	57:34 h	88.6%	54:42 h	86.8%	51:16 h	85.4%	52:56 h	89.0%	±02:24 h	±1.5%
	**0109**	56:00 h	89.6%	58:06 h	89.4%	55:18 h	87.8%	51:54 h	86.5%	53:24 h	89.7%	±02:23 h	±1.4%
	**0110**	56:30 h	90.4%	58:38 h	90.2%	55:54 h	88.7%	52:32 h	87.6%	53:52 h	90.5%	±02:22 h	±1.3%
	**0111**	57:00 h	91.2%	59:10 h	91.0%	56:30 h	89.7%	53:10 h	88.6%	54:20 h	91.3%	±02:20 h	±1.2%
	**0112**	57:30 h	92.0%	59:42 h	91.8%	57:06 h	90.6%	53:48 h	89.7%	54:48 h	92.1%	±02:19 h	±1.1%
	**0113**	58:00 h	92.8%	60:14 h	92.7%	57:42 h	91.6%	54:26 h	90.7%	55:16 h	92.9%	±02:18 h	±0.9%
	**0114**	58:30 h	93.6%	60:46 h	93.5%	58:18 h	92.5%	55:04 h	91.8%	55:44 h	93.7%	±02:18 h	±0.8%
	**0115**	59:00 h	94.4%	61:18 h	94.3%	58:54 h	93.5%	55:42 h	92.8%	56:12 h	94.5%	±02:17 h	±0.7%
	**0116**	59:30 h	95.2%	61:50 h	95.1%	59:30 h	94.4%	56:20 h	93.9%	56:40 h	95.2%	±02:16 h	±0.6%
	**0117**	60:00 h	96.0%	62:22 h	95.9%	60:06 h	95.4%	56:58 h	94.9%	57:08 h	96.0%	±02:16 h	±0.5%
	**0118**	60:30 h	96.8%	62:54 h	96.8%	60:42 h	96.3%	57:36 h	96.0%	57:36 h	96.8%	±02:16 h	±0.4%
	**0119**	61:00 h	97.6%	63:26 h	97.6%	61:18 h	97.3%	58:14 h	97.1%	58:04 h	97.6%	±02:15 h	±0.2%
	**0120**	61:30 h	98.4%	63:58 h	98.4%	61:54 h	98.3%	58:52 h	98.1%	58:32 h	98.4%	±02:15 h	±0.1%
	**0121**	62:00 h	99.2%	64:30 h	99.2%	62:30 h	99.2%	59:30 h	99.2%	59:00 h	99.2%	±02:23 h	±0.0%
**Hatch**	**0122**	62:30 h	100.0%	65:00 h	100.0%	63:00 h	100.0%	60:00 h	100.0%	59:30 h	100.0%	±02:15 h	±0.0%

Supplementary Videos

Supplementary Video 1 – Medfly embryonic development for the six *in toto* datasets (DS0001–DS0006) along four orientations from 02:00 h to 64:30 h of absolute development time with an interval of 30 min between the time points. The video starts at the onset of stage I-2, when the nuclei move to the surface of the egg and stops at the end of stage VI-17, when larvae hatch. It shows six embryogenetic events: (I) blastoderm formation, (II) early gastrulation, (III) germband elongation, (IV) germband retraction, (V) dorsal closure and (VI) muscular movement. Frame rate is five frames per second. Embryos from DS0001 and DS0003 are shown along the ventrolateral, dorsolateral, dorsolateral, and ventrolateral orientations, embryos from DS0002 and DS0004–DS0006 are shown along the ventral, lateral, dorsal, and lateral orientations. ZA, *z* maximum projection with image adjustment.
